# Tight Binding Simulation of the MgO and Mg(OH)_2_ Hydration and Carbonation Processes

**DOI:** 10.1021/acs.jctc.4c01531

**Published:** 2025-02-04

**Authors:** Jiwen Yu, Andrew Horsfield

**Affiliations:** Department of Materials and Thomas Young Centre, Imperial College London, South Kensington Campus, London SW7 2AZ, United Kingdom

## Abstract

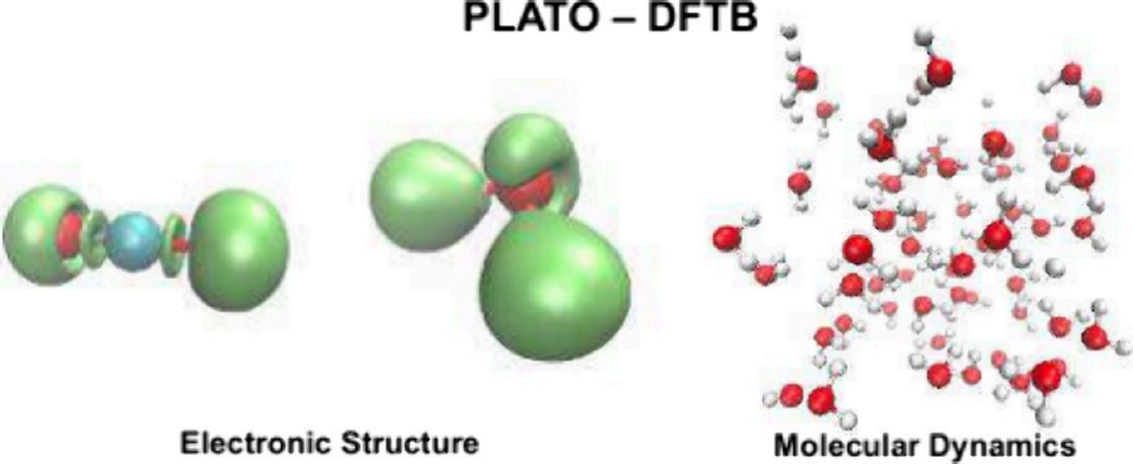

Magnesium, the lightest engineering metal, has MgO and Mg(OH)_2_ as its common corrosion products, which can also be used for CO_2_ storage due to their chemical reactivity. In this study, we developed a DFTB model with monopole, dipole, and quadrupole electrostatics for magnesium compounds containing oxygen, hydrogen, and carbon and applied it in both static and molecular dynamics (DFTB-MD) calculations of the MgO and Mg(OH)_2_ hydration and carbonation processes. With our new model, the Electron Localization Function (ELF) and Charge Density Difference (CDD) were computed as part of the electronic structure analysis, providing insights into the electronic mechanism of MgO and Mg(OH)_2_ hydration and carbonation processes. The geometry for the brucite–water bulk system was analyzed, including the reconstruction of near-surface water molecules which may influence the dissolution, hydration, and carbonation processes. By comparing experimental, DFT, classical MD results and the results from other parameter set, the accuracy of the model was assessed. A strong covalent bond between CO_2_ and the (001) surface of MgO leads to the formation of a CO_3_ group, while no such CO_3_ group forms on the (101̅1) surface of Mg(OH)_2_. Defect sites, however, are more favorable for the formation of the CO_3_ group. In contrast, covalent bonds are not found for either surface when water interacted with them. This work provides new insights into the behavior of magnesium compounds interacting with water and carbon dioxide using our model, and it introduces a tool for effectively analyzing chemical electronic structures and bonding mechanisms.

## Introduction

Magnesium, the lightest engineering metal, is widely used due to its favorable properties. Magnesium oxide (MgO) and brucite (Mg(OH)_2_), which are the primary corrosion products of magnesium, can interact with environment components such as H_2_O and CO_2_, playing an important role in corrosion process. Moreover, magnesium oxide plays a crucial role in carbon capture technologies due to its high reactivity with CO_2_. Therefore, an appropriate method is necessary to understand the mechanism of key processes, such as carbonation and hydration.

Density functional theory (DFT)^1^ is a widely adopted method for studying the physical and chemical properties of materials, but it is computationally expensive. It often requires substantial computational resources, including dozens to hundreds of CPU cores and significant memory, to calculate the properties of a large system. Molecular dynamics based on DFT (DFT-MD) further intensifies the computational demands. It has been reported that ∼50 000 CPU hours were needed for a 5 ps (0.25 fs step) calculation of a carbon cluster with 256 atoms, equivalent to running on 64 cores/128 threads for 2 weeks.^2^ Recently some groups^3^ have begun using GPUs to accelerate DFT-MD calculation with some simplifications to make it an order of magnitude faster than using a CPU, but the computational cost remains high. Classical molecular dynamics with force fields is computationally much less expensive than DFT, but it lacks the ability to provide electronic properties such as charge density distribution. Density functional tight binding (DFTB) models combine the advantages of DFT and classical molecular dynamics: it can provide electronic structure as it is a quantum method, while it can also provide geometry information at various temperatures more efficiently than DFT. Using DFTB enables MD calculations for systems containing several hundred atoms to be performed on a personal computer.

In the 1950s, Slater and Koster^4^ developed the tight binding model, a simplified quantum mechanical approach, to describe the electronic structure of materials. Electrons are assumed to be tightly bound to individual atoms and have limited interactions with other atoms. Between the 1980s and 1990s, Porezag, Frauenheim, Köhler, Seifert, and Kaschner^5,6^ used the discovery^7−9^ that the tight binding model can be directly derived from a Taylor expansion of DFT including electron exchange and correlation to develop the theoretical foundation of DFTB. The minimal set of atomic orbitals can provide a sufficiently accurate approximation of DFT for material properties and enable a high calculation efficiency. Subsequently, based on the higher order Taylor expansion of the energy functional, the second-order self-consistent DFTB (DFTB2)^10^ and third-order self-consistent DFTB (DFTB3)^11−13^ were developed to further improve accuracy in electronic structure predictions. To enhance the accuracy of long-range interaction such as hydrogen bonding, dispersion corrections^14,15^ are commonly incorporated to improve the structural predictions. Moreover, it has been reported in 2023 that van der Heide et al.^16^ introduced hybrid functional into DFTB. Exact HF exchange is calculated, and it significantly improved the accuracy of electronic structure calculated by DFTB.

In addition to the DFTB method, Grimme et al.^17−19^ developed extended tight-binding approaches GFN1-xTB and GFN2-xTB by introducing a semiempirical expression for Hamiltonian and repulsive potentials. Additionally, Wannier transformations^20,21^ are widely employed to derive parameters in the tight binding model in electronic structure analysis.

DFTB parameters are typically specifically fitted to particular systems and chemical environments. Currently, the parameter sets covering the H–C–O–Mg system include 3ob,^13,22^ the parameter set generated by Wahiduzzaman et al.^23−25^ and the PTBP parameter set generated by Cui et al.^26^ The 3ob parameter set was specifically designed for bio- and organic molecules in DFTB3 and the parameter sets developed by Wahiduzzaman and Cui are general DFTB parameter sets across the periodic table. Wahiduzzaman’s parameter set emphasizes electronic structure accuracy, while Cui’s set focuses on the energetic and structural properties of simple crystals. In the context of material science, available DFTB parameters include nanotube-related magnesium hydroxide structure (matsci-magsil),^27−29^ a magnesium–zinc system,^22^ and metallic magnesium.^30^ In this work, we present a DFTB model for understanding the hydration and carbonation processes of magnesium compounds and analyze the structural and electronic properties of MgO and Mg(OH)_2_.

DFTB is an efficient quantum method but offers lower accuracy compared with DFT, which makes it a suitable tool for qualitative analysis. Approximate parameters can be used to analyze the electron structure. The Electron Localization Function (ELF), developed by Becke and Edgecombe,^31^ is a useful parameter for measuring the electrons localization behavior. It is defined in terms of the probability of finding two electrons with the same spin at a specific point in a multielectron system. The expression used to compute the ELF can also be interpreted as the difference in kinetic energy density between the many-electron system and a single electron with an equivalent charge density. ELF is an effective tool for qualitative analysis, as its isosurface shapes can provide information about chemical bonds and electron localization regions. Certain quantities, such as the adsorption energy of a hydrogen atom, can be correlated to the ELF value at specific positions.^32^ However, using ELF to predict adsorption sites requires precise quantitative analysis, and so it has not been used in this way in this work: it is being used solely as an analytic tool instead.

In the Theory and Computational Method section, more details about DFTB are provided, and ELF and other quantities used are introduced. The results for structure calculations, reactions with molecules, and the conclusions can be found in the Results and Discussion, and Conclusions parts.

## Theory and Computational Method

### DFTB Theory

The Harris–Foulkes functional^7,9^ is the starting point of the tight binding calculation. The total energy of DFTB can be derived from a Taylor series expansion around a chosen reference density *n*^(0)^(*r⃗*), and the difference between the reference density and actual density *n*(*r⃗*) is the density fluctuation term *q*(*r⃗*).
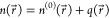
1

Equation 2 shows the total DFTB energy expanded to second order around a reference density:
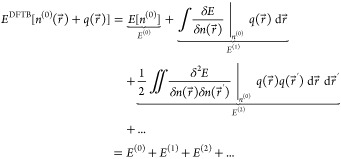
2DFTB is based on the ansatz of a Linear Combination of Atomic Orbitals (LCAO) for the molecular orbitals, which gives
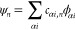
3where ψ_*n*_ is the molecular orbital for state *n* and ϕ_α*i*_ is the atomic orbital *i* of atom α. The actual density and the reference density can thus be written as
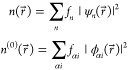
4where *f*_*n*_ and *f*_α*i*_ are the molecular and atomic orbital occupation numbers, respectively.

If we neglect second order and higher terms in eq 2, then minimizing the total energy with respect to the coefficients *c*_α*i*,*n*_, while preserving the orthonormality of the molecular orbitals, leads to an eigenvalue problem:
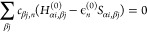
5The indices α and β refer to atoms, *i* and *j* refer to atomic orbitals. The matrix *H*_α*i*, β*j*_^(0)^ includes the hopping integral and onsite terms, while *S*_α*i*,β*j*_ is the matrix of overlap integrals. The methods to obtain the value of the elements of these matrices will be introduced in the next part.

Now we can write the expression for *E*^(1)^ in terms of the Kohn–Sham orbitals:
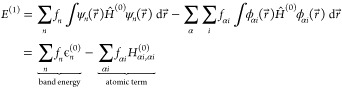
6The band energy is influenced by the hopping, overlap, and crystal field integrals. The atomic term is influenced by the onsite and crystal field energies. Since the crystal field term always contributes an attractive interaction, it often causes atoms to adhere to each other rather strongly, with the energy becoming increasingly negative as the atoms approach each other. Since this phenomenon is hard to control the crystal field term is ignored and its contribution is corrected through the pair potential in *E*^(0)^. Therefore, the atomic terms in *E*^(1)^ each become equal to the energy of an isolated atom in a vacuum.

We now consider *E*^(2)^ in eq 2, using the scheme of Boleininger et al.^33^ To begin with, we ignore the contribution from the second derivative of the exchange-correlation energy and only consider the Hartree term. So, *E*^(2)^ can be written as
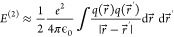
7where *e* is the charge on an electron, and ϵ_0_ is the permittivity of free space. The charge density fluctuation can always be written as the sum of atomic contributions *q*(*r⃗*) = ∑_α_*q*_α_(*r⃗*), and each atomic contribution can be written, in turn, as a multipole expansion:
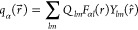
8where *Q*_*lm*_ is a charge density moment, *Y*_*lm*_ is a spherical harmonic, and *F*_*αl*_ is a radial function.^33^ We approximate the radial function by a Gaussian function as follows:^33^
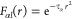
The parameter τ_α_ varies with the type of element, but not the value of *l*. It is related to the Hubbard parameter *U*_α_ of atom α by . Note that calculations of *U*_α_ usually include exchange and correlation, which thus is reintroduced into the model. The final expression for *E*^(2)^ can now be written as
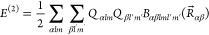
9where
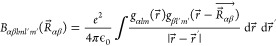
10and *B*_αβ*lml*′ *m*′_(*R⃗*_αβ_) is known as the Madelung structure constant. Here, *g*_α*lm*_(*r⃗*) = *F*_α*l*_(*r*) *Y*_*lm*_(*r̂*), and *R⃗*_αβ_ is the displacement vector between atoms α and β. The matrix *B*_αβ*lml*′ *m*′_(*R⃗*_αβ_) can be precalculated and saved as a table.

If we now minimize the total energy with respect to the coefficients *c*_α*i*,*n*_, while preserving the orthonormality of the molecular orbitals, we again obtain an eigenvalue problem, but there is now an additional term in the Hamiltonian matrix δ*H*_α*i*β*j*_ that depends on *q*:
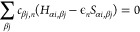
11The matrix *H*_α*i*,β*j*_ can be calculated from *H*_α′ *i*′,α*i*_ = *H*_α′*i*′,α*i*_^(0)^ + δ*H*_α*i*β*j*_. In this project, the multipole expansion includes monopole, dipole, and quadrupole, which correspond to *l* = 0, 1, and 2, respectively. More information can be found in the work of Boleininger et al.^33^

Now let us go back to *E*^(0)^. Equation 12 shows the expression for *E*^(0)^, which is the sum of isolated atomic contributions (*E*_α_^atom^), a pair potential representing the attraction and repulsion between atoms , and a many-body exchange and correlation related part (*E*^MB^_XC_):
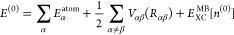
12The exchange-correlation part can be precalculated by using a multicenter expansion of the exchange-correlation energy, and the pairwise contribution can be incorporated into the pair potential *V*_αβ_: more information can be found in the work of Fogarty et al.^30^ At short range, the repulsive pair potential dominates *E*^(0)^.

### Orbital and Integral Generation

To compute the hopping and overlap integrals, we first need to calculate the pseudoatomic orbitals using DFT. An additional confining potential is added to the atom calculation to restrict the range of the orbitals. The form of the confining potential *V*_confine_ is
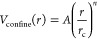
13where *A* sets the energy scale, *n* sets the steepness of the potential, and *r*_c_ is the confinement radius. The values chosen for *A* and *n* are usually 1 Ry and 2, respectively, and *A* can effectively be modified by changing the confinement radius *r*_c_, which is usually set to twice the covalent radius of the atom.^5^ After obtaining the pseudoatom orbitals ϕ_α*i*_, the onsite energy (ϵ_α*i*_), hopping integrals (*H*_α*i*β*j*_^(0)^) and overlap integrals (*S*_α*i*β*j*_) can be calculated by
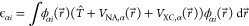
14
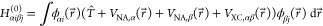
15
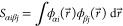
16where *T̂* is the kinetic operator, *V*_NA,α_ the neutral atom potential for site α,*V*_XC,α_ the electron exchange-correlation potential for site α, and *V*_XC,αβ_ the electron exchange-correlation potential for a pair of sites α and β. The exchange-correlation potential for a pair of sites is not just the sum of the potentials for the two sites, because of the strongly nonlinear form of the potential.

The relationship between the energy of an atom and charge, *Q* is shown in eq 17. *E*^neutral^ is the energy for neutral atom α and *U*_α_ is the Hubbard parameter for site α. Therefore, one method to calculate the value of *U*_α_ is fitting a parabola for the DFT energy with different charges and then calculating its second derivative. The Hubbard parameter can also be calculated by finding the difference between the atomic ionization energy (*IE*(α)) and electron affinity (*EA*(α)) by eq 18. In this project, the second method is adopted.
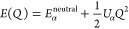
17
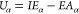
18

The onsite energies and Hubbard parameter are stored as individual numbers, while the hopping integrals, overlap integrals, and Madelung structure constants for different separations are stored in advance as a table. During a simulation, the required values can be found by performing interpolation between the values in the tables. This is one reason why the DFTB model is far faster than DFT.

### Pair Potential Fitting

The pair potential between two atoms is defined as the difference between the DFT energy and the DFTB electronic energy (*E*^(1)^ + *E*^(2)^) for those atoms. Note that the pair potential is assigned a cutoff radius, after which it is set to be zero. In practice, the derivative of the pair potential is typically fitted as the optimized structure is only related to the force or the gradient of the energy. The pair potential can be calculated by evaluating the energy gradient. Note that there is no missing arbitrary constant as the potential must go to zero at large distance. In a crystal, the overall contribution from the pair potential is the sum of pair potentials contributed by all atom pairs.

By changing the position of an atom or a group of atoms, one can obtain the gradient of the energy can be obtained. Equation 19 shows the equations that underpin the pair potential calculation process. The pair potential is divided into two components, where one can be held fixed, and the other we must optimize. For example, if we increase the bond length between a Mg atom and a hydroxide group in brucite along the (001) direction, the pair potentials for O–H and O–O remain constant because there are no long-range components of O–H and O–O inside the cutoff radius. Thus, only the Mg–O pair potential is affected. In this way, we can focus on one pair of elements at a time and obtain the pair potential for crystals.^34^
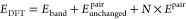
19
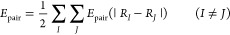
20By this method, the structure can be optimized at 0 K perfectly. However, there is still a limitation to this method: during an MD simulation, the structure may melt at an unexpected temperature. We resolved this problem by adding a correction to the pair potential. The whole pair potential is moved downward in energy, and an extra part is introduced after the cutoff to make the function converge to zero. The operation will not influence the optimized structure at 0 K because the gradient of the pair potential becomes positive after a cutoff radius, and the band energy of a system will decrease when atom separations increase: together these ensure that the total energy will not be lower than the equilibrium value.

As this approach focuses on the short-range part of the pair potential, it can introduce inaccuracies in the long-range interactions, leading to geometric errors in dimers or clusters. Therefore, additional corrections are necessary. In principle, this might be achieved by changing the confinement parameter or Hubbard parameter as well, but that would be complex to achieve as there will be very many combinations of those parameters. Instead, we employ the iterative Boltzmann inversion (IBI) method (also known as reverse Monte Carlo scheme).^35−38^ The IBI method is defined in eq 21.
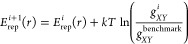
21where *i* is the iteration number, and *g* is the radial distribution function (RDF). The subscripts *X* and *Y* represent the species of the element and can be the same. The RDF can be calculated from an MD simulation using DFTB (DFTB-MD). By comparing the results with experiment or some reliable MD value (benchmark), we can obtain the corrected pair potential can be obtained. When the value of the RDF at position r is larger than the reference value, the pair potential will be made larger (more positive), so atoms will be less likely to reside at this separation and vice versa.

### Charge Density Difference

The charge density difference (CDD) is defined as
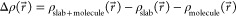
22where ρ_slab+molecule_*(r⃗)* is the charge density of the slab with a molecule adsorbed, and ρ_slab_(*r⃗*) and ρ_molecule_ (*r⃗*) are the charge density of the slab and molecule individually. The difference in charge density can indicate charge transfer during the reaction. A negative CDD value at a specific position indicates that electrons move away from this point to the surrounding environment. Conversely, a positive CDD value suggests that electrons accumulate at this point from the surrounding environment. During the study of a molecule adsorbed on the surface, the CDD can indicate that either the molecule is a charge donor or acceptor and highlight the electron rearrangement occurring during the reaction.

### Electron Localization Function

The Electron Localization Function (ELF)^31,39^ is defined as
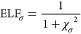
23and
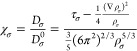
24Here, σ represents the electron spin. *D*_σ_ represents the Pauli repulsion between two electrons with the same spin σ. τ_σ_ is the kinetic energy density for electrons computed directly from the eigenstates: τ_σ_ = ∑_*n*_ |∇Ψ_*n*_ |^2^, where *n* runs over all filled energy levels. In general, , where ρ_σ_ is the charge density from electrons with spin σ. *D*_σ_^0^ represents the uniform electron gas kinetic energy density for electrons with spin σ. If the spin is not considered in the calculation, the spin density would be replaced by half of the total density. The uniform electron kinetic energy should be multiplied by a coefficient  and the coefficient in the numerator will become , as the density for the electrons with spin up should be the same as for the electrons with spin down, with each having a density equal to half the total electron density. In this case, *D*_σ_^0^ becomes , where the coefficient is termed the Fermi constant.

In summary, magnetic materials exhibit two distinct ELF values: one for spin-up and another for spin-down electrons, while nonmagnetic materials or materials where spin is ignored in the calculation have two equal ELF values or a single total ELF value. The quantity χ_σ_ is a dimensionless measure of electron localization. Since the normalization factor *D*_σ_^0^ is chosen arbitrarily, the absolute ELF value is also arbitrary. With the normalization of Becke and Edgecombe^31^ (the kinetic energy density of a uniform electron gas), the value of the ELF lies in the range 0 to 1. The value of 1 indicates that the electrons are perfectly localized at the chosen position; the value of 0.5 indicates that the behavior of electrons in this area is similar to that of uniform electron gas, and the value of 0 indicates that the electron is not localized in this area. The ELF can be used to predict possible adsorption sites on the surface, for both physical and chemical bonding, and can help interpret atomic bonding mechanisms.^31,39^

In this study, both CDD and ELF are derived entirely from wave function results obtained via DFTB.

### Calculation Details

The PLATO package^40^ was used to obtain the atomic orbitals, to tabulate the hopping and overlap integrals, and to calculate Hubbard parameters. In our calculations, the electron exchange and correlation were described using the Perdew–Burke–Ernzerhof (PBE) functional^34^ and the electron–nucleus interactions were described using separable dual-space Gaussian pseudopotentials.^41−43^ For the atomic orbital calculations, the value of *r*_0_ for the confinement potential for H, C, O, and Mg was set to be 1.17*a*_0_, 2.87*a*_0_, 2.50*a*_0_ and 5.33*a*_0_ respectively, where *a*_0_ is the Bohr radius. The value of *n* for the confinement potential was set to 2. The cutoff radius for H was set to be 5*a*_0_, for C and O it was set to be 6*a*_0_ and for Mg it was set to be 7*a*_0_. The hopping integrals include the two-site approximation for exchange and correlation, while the crystal field terms were ignored. The energies of an ion with charge of +1 and an ion with charge of −1 were used to calculate the Hubbard parameter. Multipole integrals to second order (dipole) and third order (quadrupole) were calculated using eq 10.

The Quantum Espresso package was adopted for the pair potential fitting.^44^ The PBE, PBEsol,^45^ hybrid functional HSE06^46^ and HSEsol^47^ electron exchange and correlation functionals were used to calculate the reference structure and energy for pair potential fitting. The Projector Augmented-Wave (PAW) pseudopotential^48^ was adopted for energy calculation of all functionals and structure optimization of PBE and PBEsol functional, while Norm-Conserving pseudopotential^49^ was adopted for HSE06 and HSEsol structure optimization calculation, because there are compatibility issues for Quantum Espresso relaxation mode when using PAW–PP. Although the hybrid functional cannot improve the electronic structure calculated by DFTB, it can also provide a proper structure as reference and can serve as a basis for band gap comparison. In addition, the van der Waals interaction was considered by adding dispersion correction proposed by Grimme et al.^50,51^

Both static DFTB and DFTB-MD calculations are supported by the PLATO package.^52^ In addition, the comparative DFTB results by other parameter sets were calculated by DFTB+.^5,6,53^ The DFTB parameters we computed were used to calculate the geometry and electronic structure for H_2_O and CO_2_ adsorbed on the surface of MgO, brucite, and brucite with a defect on the (0001) surface. A slab formed from one 3 × 3 × 3 supercell was used to represent the MgO(001) surface, a 4 × 4 × 4 supercell for the brucite(0001) surface, and a 3 × 3 × 3 supercell for the brucite(101̅1) surface. Water and carbon dioxide molecules with different geometries were then placed at several adsorption sites on these surfaces to find the minimum energy configurations. A 3 × 3 × 1 *k*-point mesh was used in each case. Density of States (DoS) calculations are implemented in the PLATO package, while ELF, CDD and the geometrical calculations described below were performed using extra Python codes written for this project, which can be found through the GitHub link provided in the Data and Code Availability section.

To study bulk water on a brucite surface, a 4 × 4 × 5 supercell of brucite was created to form a slab, and a 12.4 Å × 10.74 Å × 15 Å water layer containing 53 water molecules was placed on the surface of the brucite. The density of the water was set to be equal to the experimental equilibrium density for the bulk water. All DFTB-MD calculations were carried out at 300 K with the NVT ensemble and temperature rescaling thermostat. Nose–Hoover thermostat is available in PLATO as well. The time step was set at 1 fs. Because the system is large, only the Γ point was used for *k*-point sampling. Once the simulations were complete, the following geometrical information was calculated: the separation between the solid surface and oxygen atoms in the water; the angle between the dipole direction of water molecules and the solid surface (this can help us understand the influence of hydrogen bonds); the angle between the normal to the plane of water molecules and that of the brucite surface (this can help us understand the adsorption geometry and interwater molecule geometry). All DFTB calculations were performed on Intel Core i5–12500 with 6 cores, which is a common CPU for household PCs.

## Results and Discussion

### DFTB Parametrization

The hopping and overlap integrals, the pair potential, and the Madelung structure constants, which are all functions of the atom separations, have been tabulated and stored in files. The parameters are available via the GitHub link provided in the Data and Code Availability section. The onsite energies and Hubbard parameters were compared with those from the parameter set generated by Wahiduzzaman,^23−25^ the 3ob parameter set,^13,22^ the PTBP parameter set generated by Cui et al.,^26^ and the matsci parameter set.^27,29^ Detailed comparisons of these parameter sets are provided in the Supporting Information. We found that there is no significant difference with our values for H, O, and C, except for the Hubbard parameter of Mg, which is 7.68 eV. Other parameter sets, except 3ob, have different values for each orbital shell. The values for the outermost *p*-orbitals from the other parameter sets are 4.10 eV (Wahiduzzaman), 6.16 eV (3ob), 7.02 eV (matsci), and 6.79 eV (PTBP). One possible reason may be that the electron affinity energy of magnesium is close to 0 eV, leading to *E*(*Q*) = 0 when *Q* is negative. This results in a reduced overall gradient of the parabola and, consequently, a lower Hubbard parameter, which corresponds to the second derivative of *E*(*Q*). For comparison, the ionization energy of magnesium is reported as 7.65 eV in the NIST database.^54^ It remains unclear whether variations in the Hubbard parameter within the same parameter set significantly impact the calculation accuracy.

The parameters obtained were utilized to compute the relaxed structures for MgO and Mg(OH)_2_ crystals, and the results were compared to those from other DFTB parameter sets. The lattice constant of MgO is 4.25 Å, while the lattice constants of Mg(OH)_2_ are *a* = 3.10 Å and *b* = 4.58 Å. As a comparison, the experiment value of MgO is 4.21 Å,^55^ while the lattice constants of Mg(OH)_2_ are *a* = 3.15 Å and *b* = 4.77 Å.^56^ It can be found that our parameter generated has a good agreement with experiment with an error of 0.95% for MgO and mean error of 2.46% for Mg(OH)_2_, and these errors are smaller than those from other DFTB parameter sets. Although the band gap of DFTB methods are closer to experiment values compared to PBE, this is merely a coincidence. In our onsite energy and hopping integral calculations, the influences of neighboring atoms are ignored. In addition, the basis in DFTB is limited so charge information will lose during calculations. Moreover, the crystal field term is also neglected, which contributes to band energy as well. These factors collectively result in narrower band widths and larger band gaps.

The surface structures of MgO and Mg(OH)_2_ were also calculated. For the MgO(001) surface, the outmost layer separation becomes smaller, and O is located 0.02 Å farther outward than Mg. This phenomenon is consistent with DFT results, but DFT gives a larger value with 0.05 Å. The electron reconstruction of the surface is interesting. According to Mulliken population analysis of the SCF2 scheme and taking the bulk Mg as the reference, the charge of Mg in the first layer is −0.12, while the charge of Mg in the second layer is +0.04. For the bulk O as the reference, the charge carried is increasing gradually. The difference between the O in the first and second layer and bulk are +0.07 and +0.01, respectively. Because the Mulliken charge is not suitable for plane wave basis, the Löwdin charge was adopted for charge analysis in DFT. Normally, the value of the Löwdin charge will be smaller than that of the Mulliken charge. As a comparison, the charge differences calculated by Löwdin charge between Mg in the first layer, second layer, and bulk are −0.03 and +0.01, and for O, it is +0.01 and +0.01 by PBE DFT.

For the Mg(OH)_2_(0001) surface, the layer separation of outermost layer decreases by 0.002 Å, which is less than that of PBE-DFT value which is 0.015 Å but similar to 0.002 by PBEsol-DFT. The underestimation is also found in other DFTB parameter sets. To fit a perfect parameter set considering all aspects of Mg(OH)_2_ is still challenging, and the interlayer distance still cannot be perfectly described. For the Mg(OH)_2_(101̅1) surface, there are two types of OH groups on the (101̅1), one is the OH group without breaking Mg–O bond and connecting with 3 Mg atoms, and the other is the OH group connecting with 2 Mg atoms and one Mg–O bond breaks. The orientation of the OH group with 2 Mg remains almost unchanged, while the orientation of the other type of outermost OH group moves outward. Based on Mulliken analysis, the charge difference of these two types of O is only 0.01, while the differences between two types of surface O and bulk O are 0.01 and 0.02, respectively. The differences are far less than that of the O group in MgO, and it reveals that the chemical environments of those OH groups in Mg(OH)_2_ are similar. This can be also verified by ELF, which can be found in the next part.

**Figure 1:**
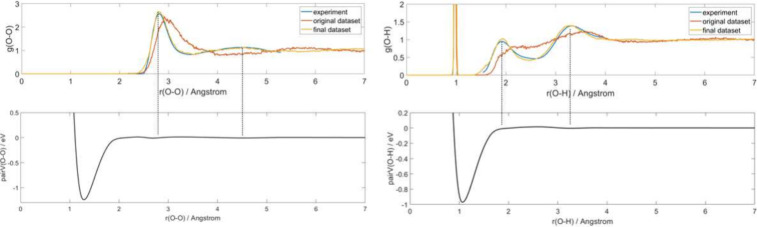
Radial distribution functions (RDFs) of O–O and O–H for water at room temperature. Experimental values,^37,38,57,58^ results from the original parameter set, and results from the parameter set after 5 iterations of the IBI correction method are shown, as well as the relevant pair potential.

The long-range parts of the pair potentials between O and O, and between O and H were fitted to experimental radial distribution functions for water.^37,38,57,58^Figure 1 shows the radial distribution function from experiment as a benchmark, along with those from our original parameter set and the parameter set after 5 iterations of the IBI correction method as well as the corresponding pair potential. The fitting of the long-range part of the pair potential significantly improves accuracy. Fluctuations also occur at the peak of RDF with a maximum value of 0.015 eV, which reveals that even a small change in long-range pair potential can lead to significant changes in configurations. Goyal et al.^37^ modified the 3ob parameter set using this method as well and presented their pair potential in their publication. The main difference between our pair potentials and theirs is that they added a repulsive pair potential in the long-range part and then applied the IBI method, while ours does not have the first step. The final results show that both represent a considerable degree of accuracy.

The long-range correction to the C–O pair potential can be fitted in the same way. One CO_2_ molecule can be placed in bulk water and the RDFs of C–O and C–H can be calculated and substituted back into eq 21 to update the pair potential. However, most of the RDF data for C–O are generated by molecular dynamics calculations and there are differences among their results.^59,60^ Therefore, it is difficult to find a good benchmark. There appears to be a consensus that for the C(CO_2_)–O(H_2_O) distance the first RDF peak appears at ∼4 Å. Our parameter set also gives the first RDF peak at ∼4 Å; therefore, no further IBI correction is added to carbon.

### The Influence of Introducing Dipole and Quadrupole Terms

Several structures including bulk MgO, MgO with a surface, and water molecules were calculated by SCF1, SCF2, and SCF3 schemes to present the influence of the multipole terms in *E*^(2)^. We found that introducing dipole and quadrupole terms in *E*^(2)^ has a minimal influence on MgO, because the MgO crystal is not polarized and exhibits high charge symmetry. However, for polarized molecules and the structure with surface that disrupts the local symmetry, introducing dipole and quadrupole terms will make significant changes to the energies of molecular orbitals and DoS of crystals with a surface. It should be noted that the dipole and quadrupole terms will introduce changes to the energies and forces. As a result, the pair potential was fitted using monopole charges only, which may lead to reduced accuracy of geometric structure when higher-order terms are included.

For single water molecule calculations, introducing dipole and quadrupole terms has a significant influence on the HOMO–LUMO, LUMO–(LUMO–1), and (LUMO–1)–(LUMO–2) energy gaps. The HOMO–LUMO gap varies from 15.31 eV (SCF1) to 14.79 eV (SCF2) and 14.92 eV (SCF3). The gap between HOMO and HOMO–1 becomes smaller on going from 1.47 eV (SCF1) to 1.38 eV (SCF2). While the gap between HOMO–1 and HOMO–2 becomes larger going from 1.60 eV (SCF1) to 1.84 eV (SCF2), showing improved agreement with other computational methods,^61^ which suggest that the energy levels of HOMO and HOMO–1 should be close. However, the gap between LUMO and LUMO+1 is not improved. In comparison, the experimental value of the HOMO–LUMO gap is 8.09 eV, and the gap between HOMO–1 and HOMO is 1.65 eV.^62^ The introduction of multipoles partially alleviates the issue of gap overestimation by the LCAO ansatz; however, the effect is limited.

**Figure 2:**
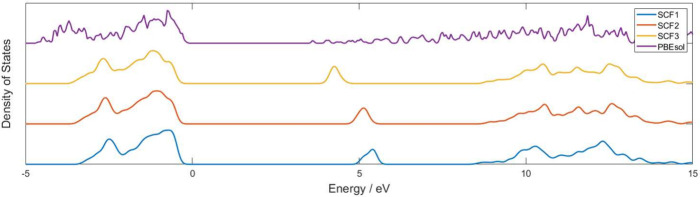
Density of states of MgO with (001) surface calculated by SCF1, SCF2, SCF3, and DFT-PBEsol.

Figure 2 shows the density of states of the MgO(001) surface calculated by three DFTB self-consistent schemes and DFT with the PBEsol functional. For the MgO(001) surface, it can be found that, for all three schemes, an isolated surface state appears, but located at different energies. The band gap between valence band and surface state are 4.64 eV (SCF1), 4.48 eV (SCF2), 3.77 eV (SCF3), and 3.50 eV (PBEsol), respectively. As a comparison, Heo et al.^55^ used reflection electron energy loss spectroscopy (REELS) and high-energy resolution REELS (HR-REELS) to obtain the surface band gap of MgO, which is 6.3 eV, and the surface band gap of the bulk is 7.8 eV. The gap between the valence band and the surface state may influence the degree of adsorption. The introduction of dipole and quadrupole terms provides more peaks in both the conduction and valence bands. There is still a large difference compared with DFT. The surface states of the MgO (001) surface overlap with the conduction band according to DFT. However, the isolated nature of the surface state can make it easy to analyze the orbital hybridization.

In summary, introducing dipole and quadrupole terms partially alleviates the gap overestimation issues for polarized system caused by the LCAO ansatz, but the band gap and bandwidth are still dominated by the use of LCAO with a small basis. Considering the calculation time and the convergence problem caused by higher orders of the multipole expansion, currently only SCF1 (monopole) and SCF2 (monopole and dipole) schemes are recommended.

### Adsorption on the MgO (001) Surface

The parameters were applied to simulate the interactions between water and carbon dioxide with the MgO(001) surface to simulate the processes of brucite formation and carbonation. First, different adsorption configurations of water or carbon dioxide molecules on the MgO(001) surface were considered as the starting point. We found that both the surface energy and the adsorption energy are overestimated. The reason for overestimated adsorption may be that the surface energy is itself overestimated, which makes the bond formed with the surface more stable. It is not possible to correct the surface energy without reducing the accuracy of the other quantities. At the optimized bond length in the crystal, the overestimation of the surface energy occurs through the band energy related term. If we change the pair potential term, it will produce large errors in the energy of bulk crystals. After relaxation, the electronic structure of the system was studied, which includes ELF, CDD, and DoS. In the Supporting Information the isosurface for the ELF of CO_2_, H_2_O, and CO_3_ group in MgCO_3_ are presented as a reference, along with detailed information about the optimization starting points and the adsorption energies.

**Figure 3:**
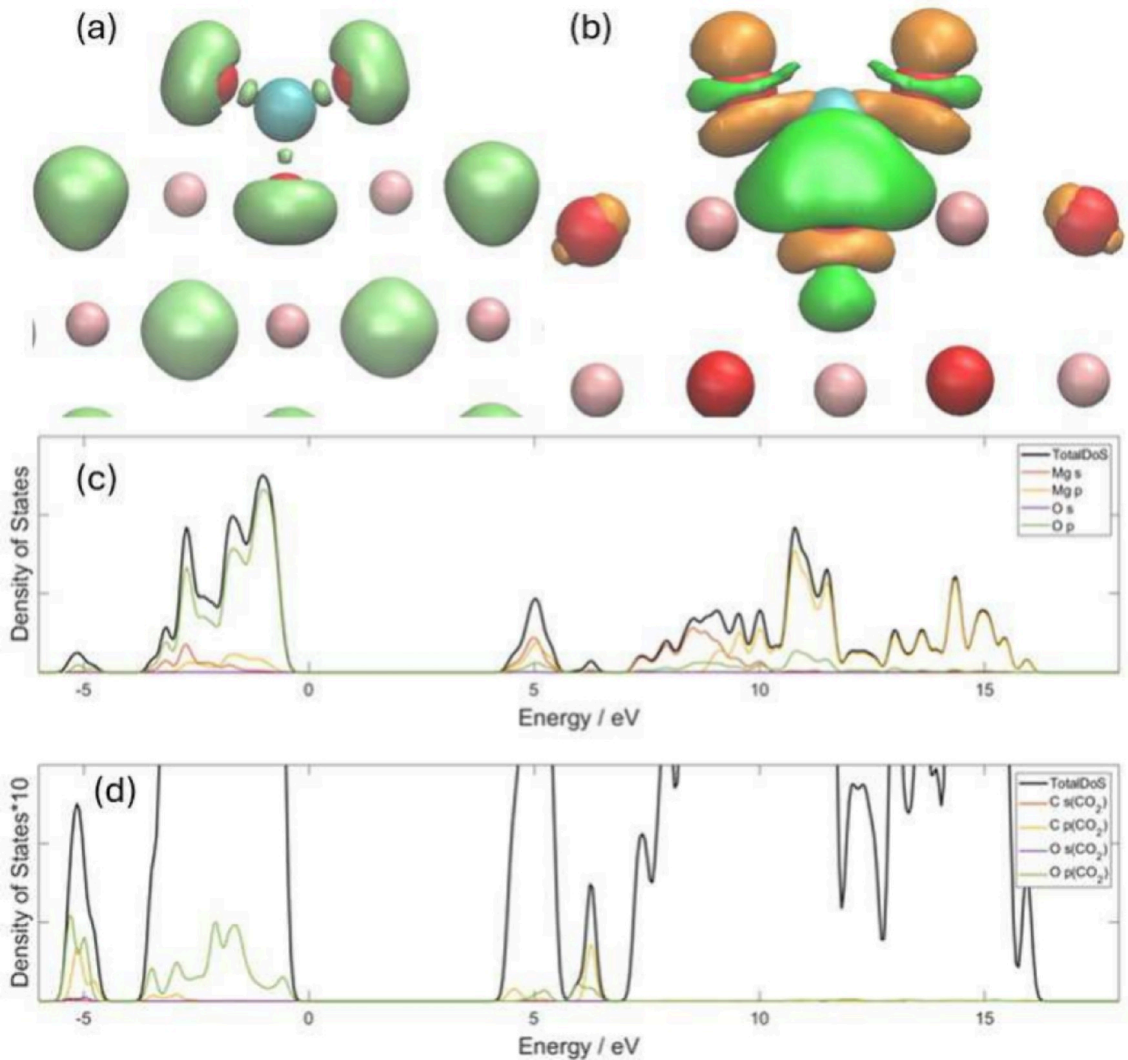
Results for the adsorption of CO_2_ on the MgO(001) surface. The coral balls represent Mg atoms, the blue ball represents the C atom, and the red balls represent O atoms. (a) The ELF isosurface with a value of 0.8, and (b) the CDD isosurface with values of ±0.025 eBohr^–3^. The orange color represents the positive value, while the green color represents the negative value. (c) The partial density of state of the MgO, and (d) the total and partial density of states of CO_2_ in the same system. The scale of panel (c) is 10 times bigger than the scale of panel (d).

Figure 3a illustrates the optimized structure and ELF of a CO_2_ molecule adsorbed on the (001) surface of MgO. The bond length between C and O in carbon dioxide increases from 1.18 Å in the gas phase to 1.25 Å. The bond length between C and the O in MgO is 1.43 Å, which is longer than the original C–O bond. The carbon dioxide bond angle becomes 135.8°. The C–O bond length from our parameters is close to the DFT results, which are 1.44 Å for the PBEsol and HSE06 functionals and 1.47 Å for the PBE functional. It was found that all three O atoms connected with the C atom have a mushroom-shaped ELF isosurface, which is the same as that in MgCO_3_ but different from other O atoms in MgO. Between the C and the O in MgO, there are areas with a high ELF value, which indicates that strong covalent bonds are formed between the carbon atom of CO_2_ and an O atom of MgO, but the bond strength is still weaker than that in CO_2_, according to the volume of the ELF isosurface. Both the mushroom-shaped isosurface and isolated ELF isosurface can be indicators of whether covalent bonds are formed between C and O.

Figure 3b shows the CDD of this system. It was found that, along the vertical direction to the surface, the electrons move to the regions on both sides of the O in CO_2_, whereas the electrons around the O in MgO undergo the opposite process. Mulliken population analysis shows that the CO_2_ molecule works as an electron acceptor and obtains 0.457 electrons from MgO, and C becomes more electron-rich, and O becomes less electron-rich, due to the elongation of the C–O bond. Both the ELF and CDD diagrams show that CO_2_ has a strong reaction with the MgO(001) surface.

Figures 3c and 3d show the partial density of states (PDoS) of this system. The states of the O atom in CO_2_ appear in both peaks shown in the valence band, which mainly have overlap with the *p* orbitals of O. The states of C mainly appear in the second peak of the valence band around −5 eV, which have strong overlap with the *p* orbital of O as well in MgO. In the area at ∼5 eV, the *p*-orbital of C and the *p*-orbitals of O in CO_2_ appear in both the first and second peaks, but mainly appear in the second peak which also have overlap with the surface states of MgO. The hybridization shown in the DoS plot also proves the strong reaction of CO_2_ with MgO.

**Figure 4:**
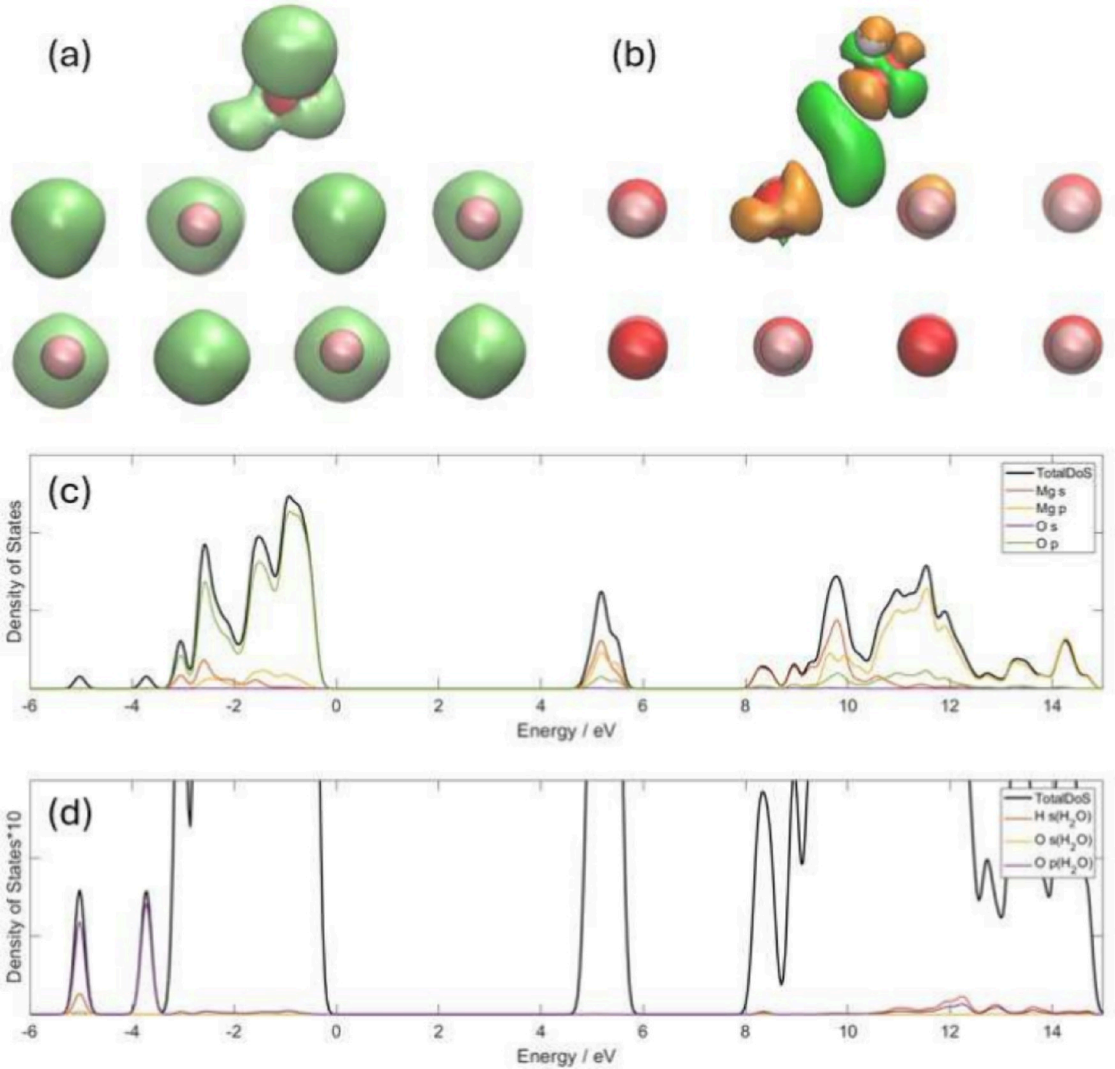
H_2_O on the MgO(001) surface. The coral balls represent Mg atoms, the red balls represent O atoms, and the white balls represent H atoms. (a) ELF with isosurface of 0.8, and (b) CDD with isosurface of ±0.013 e Bohr^–3^. The orange color represents the positive value, while the green color represents the negative value. (c) The partial density of states of Mg and O in MgO, and (d) the total and partial density of states of H_2_O in the same system. The scale of panel (c) is 10 times bigger than the scale of panel (d).

Figure 4a shows the optimized geometry and ELF for a H_2_O molecule adsorbed on the MgO(001) surface. The O–H bond which is closer to the surface increases its bond length from 0.97 Å to 1.00 Å, while the other O–H bond length remains at 0.97 Å. The separation between H in H_2_O and the O in MgO is 1.60 Å. The bond angle of the water molecule becomes 104.3°. As a comparison, the O–H bond length in brucite calculated by the same parameter is 0.97 Å. Because the adsorption of H_2_O on the surface is physical adsorption, the strength of the hydrogen bond plays an important role in the adsorption configuration and the choice of dispersion correction and long-range pair potential will be important. Our optimized structure is similar to the structure calculated by Alessio et al.^63^ using PBE DFT. The ELF isosurface around the O atom in H_2_O is slightly pushed away from the surface, and is no longer aligned along the same direction as the original dipole direction of water, but the shape keeps unchanged. Comparing the ELF graph with that of a water molecule in a vacuum, we can see that the electrons are much more localized around the H atom in the water molecule, which is close to the MgO(001) surface, and the ELF isosurface around the O atom in MgO becomes asymmetric but does not change much. This indicates that the electron environment around the O in the surface does not change much, and the adsorption is weaker.

Figure 4b shows the CDD of the same system. The electron reconstruction was found in the O atom of H_2_O. The H atom close to the surface loses electrons, while the other H atom gains electrons. According to Mulliken population analysis, even though the H_2_O molecule also works as an electron acceptor, it only obtains 0.016 electrons from MgO, which is far less than that for CO_2_. In addition, the dipole direction of the water molecules also moved upward, which is the same effect that the ELF diagram indicates. Overall, the reaction of H_2_O with the MgO(001) surface is far weaker than that of CO_2_. There is no obvious OH group generated, but one H atom gains electrons, while another loses electrons. This may reveal that a further condition needed to form a separated OH group.

Figures 4c and 4d show the projected DoS (PDoS) of this system. We can see that the *s*-orbital of H and the *s*- and *p*-orbitals of H_2_O form three isolated peaks. Only the *p*-orbital of O overlaps with MgO in the valence band, and the *s*-orbital of H has only a small overlap with the *p*-orbitals of O and Mg. There is almost no overlap with the MgO surface states. This again leads to the conclusion that the reaction between H_2_O and the MgO(001) surface is weak.

### Adsorption on Mg(OH)_2_(101̅1) Surface

The reactions between Mg(OH)_2_, water, and carbon dioxide molecules are now studied. Since it is hard for both H_2_O and CO_2_ to react with a perfect brucite(0001) surface, because of the orderly arrangement of OH groups, the (0001) surface-related properties were calculated by DFTB molecular dynamics (DFTB-MD). This gives the atoms kinetic energy, allowing them to overcome small energy barriers. The (101̅1) surface is an interesting surface because it is in contact with the solution when brucite dissolves.^64^

**Figure 5:**
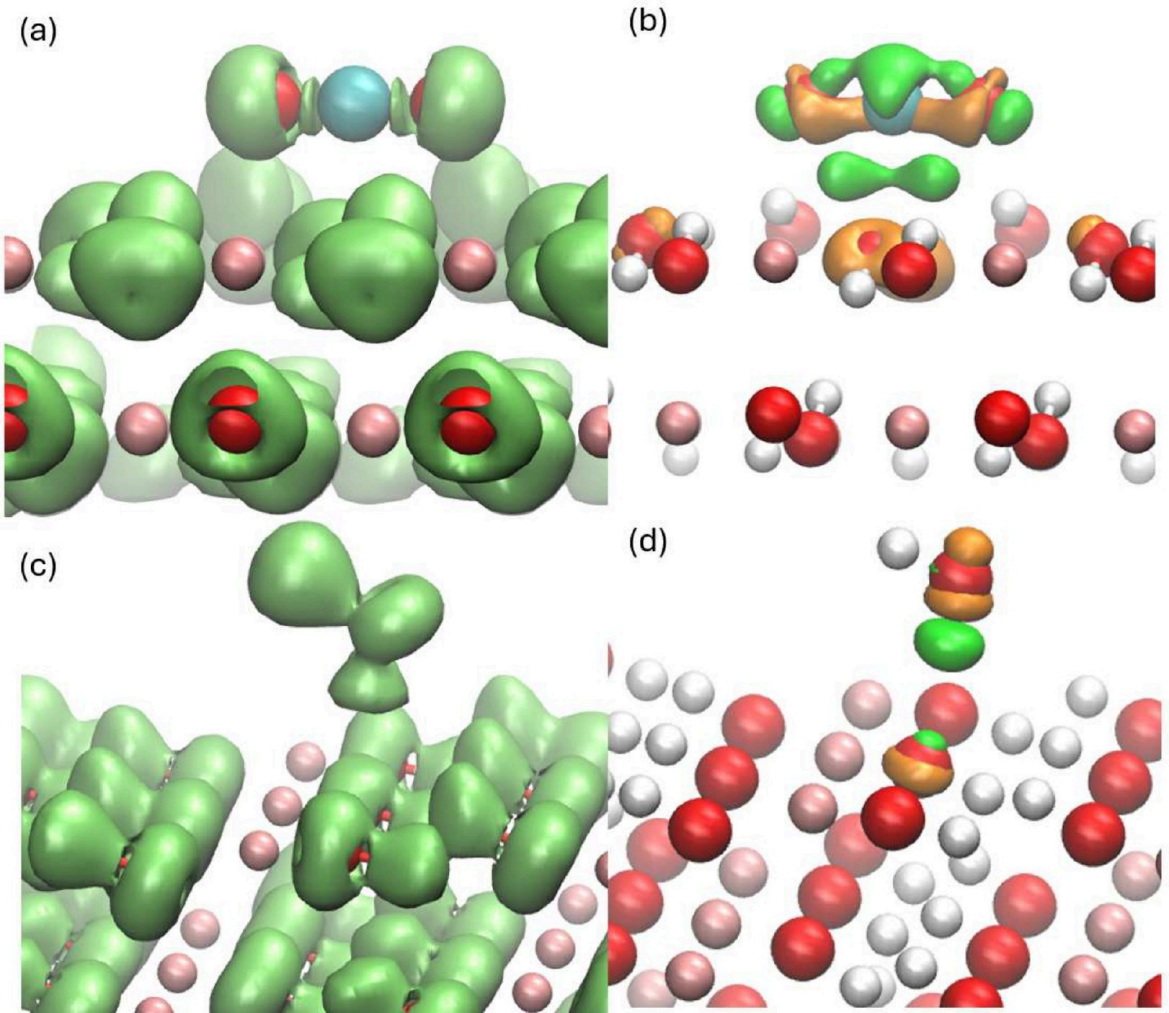
(a)The ELF with isosurface of 0.8 for optimized structure of a CO_2_ adsorbed on the Mg(OH)_2_(101̅1) surface and (b) the CDD with isosurface of 0.0015 e Bohr^–3^ for the same system; (c) The ELF with isosurface of 0.8 for optimized structure of a H_2_O adsorbed on the Mg(OH)_2_(101̅1) surface and (d) the CDD with isosurface of 0.0015 e Bohr^–3^ for the same system.

Both CO_2_ and H_2_O are physically adsorbed on the (101̅1) surface of Mg(OH)_2_. For physical adsorption, there is no valuable information available from the DoS diagram; therefore, it will not be shown here. Figures 5a and 5b show the optimized structure, ELF, and CDD of CO_2_ adsorbed on the Mg(OH)_2_(101̅1) surface. The O atoms in CO_2_ are over the Mg atoms on the (101̅1) surface, and the separation between the CO_2_ molecules and the (101̅1) surface is 2.18 Å. The separations calculated by different functionals in DFT have differences, and they range from 2.8 Å to 3.1 Å. The difference between the DFTB and DFT values may be improved by further fitting the long-range C–O and Mg–O pair potentials. However, our DFTB model correctly identifies the adsorption as physical adsorptions. It is found that the ELF isosurface of CO_2_ is almost the same as that under vacuum with only minor deflections generated due to the surface. CO_2_ still works as an electron acceptor and obtains only 0.0038 electrons, according to Mulliken population analysis. This can be shown using CDD as well, where a clear isosurface can be found only when the value is set as low as 0.0015 e Bohr^–3^.

Figures 5c and 5d show the optimized structure, ELF, and CDD of H_2_O adsorbed on the Mg(OH)_2_(101̅1) surface. The separation between the H atom in H_2_O and the O atom on the Mg(OH)_2_(101̅1) surface is 1.82 Å. The O–H bond, which is closer to the surface, only increases from 0.97 Å to 0.98 Å, while the other O–H bond remains at 0.97 Å. The ELF isosurface shape of H_2_O is similar to that for H_2_O adsorbed on the MgO(001) surface. The isosurface is pushed away from the surface and is no longer aligned along the original direction. The change in the ELF isosurface shape of H_2_O can also reveal the change of the dipole direction of the H_2_O adsorbed on the surface. For the OH group, there is almost no change of its ELF isosurface shape. The H_2_O molecule works as an electron acceptor but only obtains 0.02 electrons according to Mulliken population analysis. CDD shows that the H atom which is closer to the surface loses electrons but the amount is negligible.

**Figure 6:**
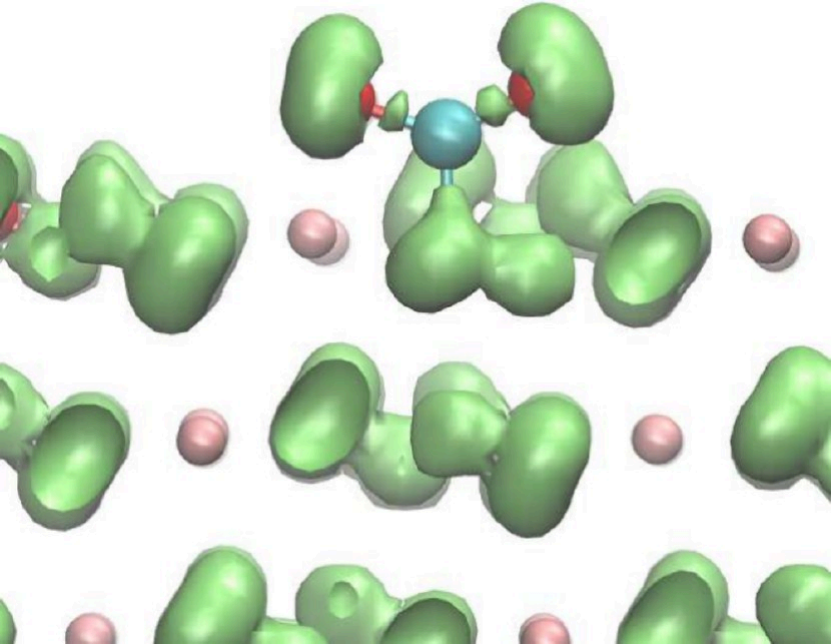
ELF with isosurface of 0.8 for optimized structure of a CO_2_ adsorbed on the Mg(OH)_2_(101̅1) surface with our previous parameter set. A similar structure can be obtained using the PTBP parameter set. However, this structure is not observed in the relaxed structure from DFT calculations.

During the fitting, the use of inappropriate weightings for different reference structures led to the appearance of structures similar to that of CO_3_, which cannot be observed in DFT calculation. This issue can also be observed in other general parameter sets, such as PTBP, which is likely caused for the same reason. Figure 6 shows the ELF structure of CO_2_ interacting with an OH group using one of our earlier parameter sets. It can be observed that, although a CO_3_ group is formed due to the large pair potential, neither the isolated ELF isosurface between the C and O atoms on the Mg(OH)_2_(101̅1) surface nor the mushroom ELF shape were found around the O atom, which means there is no strong covalent bond formed in this case. The ELF shape of the O atom in OH group is still similar to other OH groups. This shows that DFTB not only relaxes the geometry but also provides appropriate electronic structure information for chemical reaction analysis.

### Adsorption on Defective Mg(OH)_2_(0001) Surface

**Figure 7:**
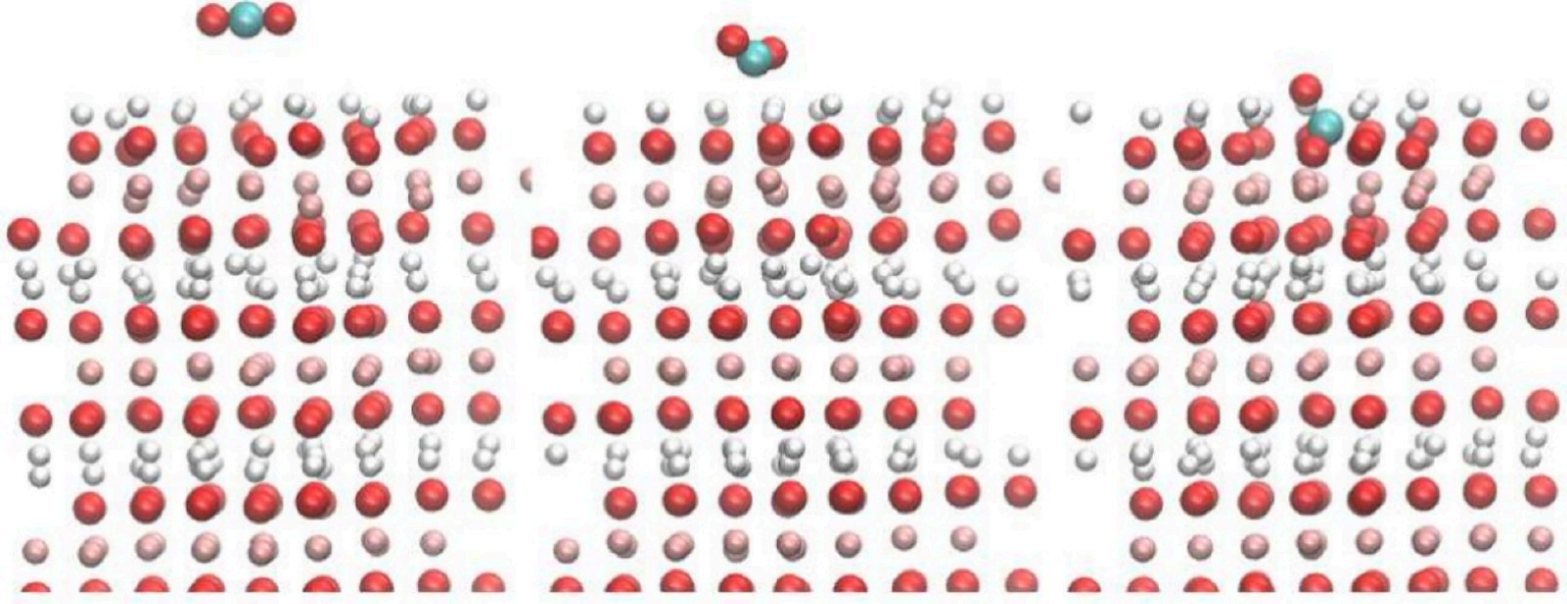
Three snapshots of a CO_2_ molecule adsorbed by the defect sites on the Mg(OH)_2_(0001) surface.

We now study the influence of a surface defect on the carbonation process of brucite. Because the complexity of the system with a defect is far greater than that without, MD calculations have become a good choice to screen the lowest energy configuration. MD allows the system to be optimized to a relatively lower energy configuration, as the kinetic energy of the nuclei allows low energy barriers to be overcome. First, an OH group and an H atom at an adjacent OH group were removed and a CO_2_ molecule was placed over the defect site. After the CO_2_ molecule overcame any energy barriers and formed a covalent bond at the defect site, a relaxation calculation to the lowest energy state was carried. Figure 7 shows three representative snapshots of a CO_2_ molecule adsorbed by the defective (0001) surface of Mg(OH)_2_. The video of this process can be found in the Supporting Information. It was found that the CO_2_ molecule was first physically adsorbed by the (0001) surface and then embedded in the defect sites.

**Figure 8:**
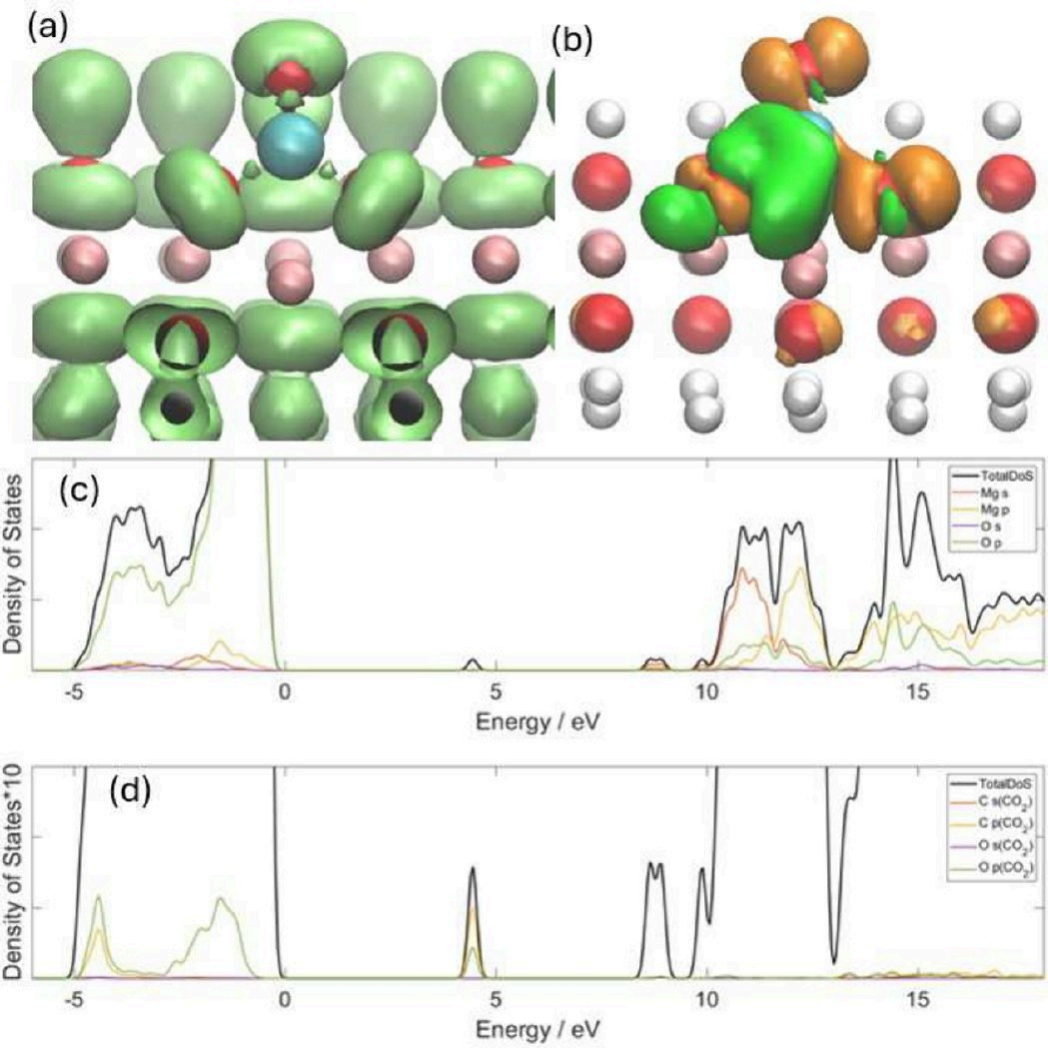
CO_2_ on the brucite(101̅1) surface with a defect site. The coral balls represent Mg atoms, the blue ball represents the C atom, the red balls represent O atoms, and white balls represent H atoms. (a) ELF with isosurface of 0.8. (b) the CDD with isosurface of ±0.025 e Bohr^–3^. The orange color represents the positive value, while the green color represents the negative value. (c) the total and partial densities of states of Mg, O and H in brucite with CO_2_ adsorbed in the defect site, and (d) the total and partial densities of states for CO_2_ in the same system. The scale of panel (d) is 10 times greater that of the scale of panel (c).

Figure 8a shows the relaxed structure and ELF of a CO_2_ molecule adsorbed in the defective Mg(OH)_2_(0001) surface. The upper C–O bond length in CO_2_ is 1.23 Å, while the other C–O bond, which is closer to a Mg atom, has a length of 1.37 Å. The bond angle of CO_2_ becomes 122.3°, which is close to the 120° of MgCO_3_. There is reconstruction in the first layer of brucite as well, with the Mg atoms around the CO_2_ molecule being pushed into the crystal. All three O connected with C have mushroom-shaped ELF isosurfaces. The isolated isosurface area of the upper C–O bond is larger than those of the other two C–O bonds, which is consistent with the bond length order.

Figure 8b shows the CDD of this system. The CO_2_ molecule works as an electron acceptor and obtains 0.561 electrons from the brucite, which is similar to MgO according to Mulliken population analysis. In addition, the O atom in the CO_2_ which is closer to the Mg atom is more negative than the other atom, leading to a large dipole moment or strong polarization in the CO_2_. The CDD shape is quite similar to that in MgO and the direction rotates through ∼60°, but shows a significant shift toward the interior direction of the crystal.

Figures 8c and 8d show the DoS values for this system. It can be found that the *p*-orbital of C and the *p*-orbital of O overlap with the *p*-orbital of O in the defect state, and there is a large overlap between the *p*-orbital of C and the *p*-orbital of O in a wider energy range than that for MgO in both valence and conduction bands. This also indicates that the chemical properties of the two O atoms in CO_2_ are different and contribute to different energy levels, making the overlap with the *p*-orbital of C greater.

We found it difficult for CO_2_ to form covalent bonds on the (101̅1) surface and can be physically adsorbed. However, it can form a covalent bond on the defective (0001) surface that is even stronger than with the MgO(001) surface. The chemical environment around the defect site plays an important role in facilitating the adsorption of CO_2_ in the defect site.

### DFTB-MD of Mg(OH)_2_(0001) Surface with Bulk Water

**Figure 9:**
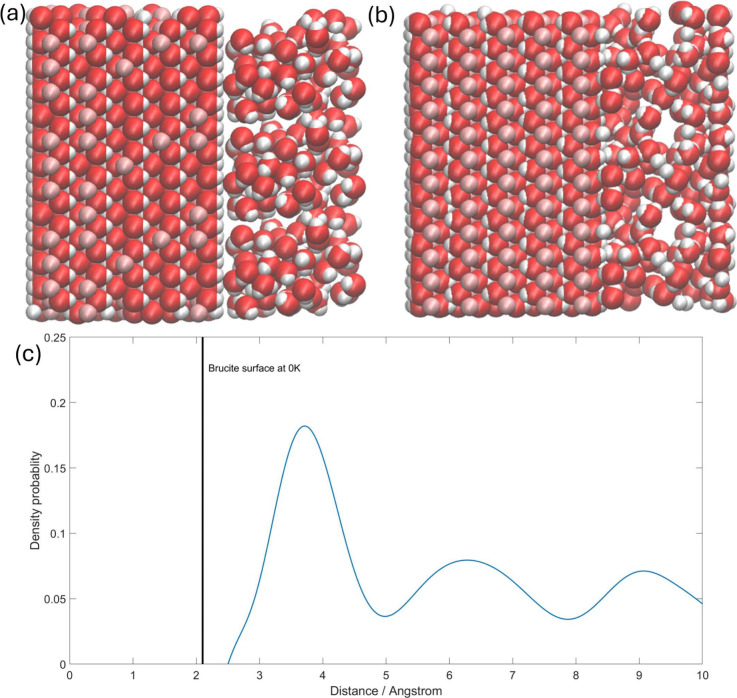
A brucite slab in contact with bulk water. The coral balls represent the Mg atom, the red balls represent O atoms, and the white balls represent H atoms. The geometry of (a) the starting point, (b) a later representative snapshot of the brucite-water system, and (c) the distance between O in water molecules and the average *z*-position of Mg. The equilibrium surface of brucite is labeled on the graph. A distance of 10 Å is near the midpoint of the water bulk, and the other side is approximately symmetric to this graph. The curve is fitted and smoothed.

In our calculations, we have assumed there is vacuum next to the surface of the brucite, but in reality, an aqueous solution environment is the most common scenario. A randomly distributed collection of water molecules representing bulk water was placed above the surface of brucite(0001) surface. The geometrical properties of this system were studied after 3 ps of MD simulation. The total energy of the system decreases rapidly and fluctuates to reach a thermal equilibrium after 50 fs. Figure 9a shows the starting point of the DFTB-MD, and Figure 9b shows one representative snapshot of the DFTB-MD calculation. Figure 9c shows the probability density of finding an O atom at a certain distance from the average position of the Mg atoms on the surface. Since the OH group will change its direction during an MD simulation, it will be hard to find a certain plain surface of brucite against which to measure; therefore, the plane of the Mg atoms on the surface is a good choice for measuring this distance. The equilibrium surface at 0 K is labeled on the graph as well. It can be found that water molecules are adsorbed by the brucite(0001) surface and form a dense water layer ∼4 Å above the Mg plane, which is the first and tallest peak in Figure 9c. The second and third peaks are of similar height and are located at 6.2 and 9.1 Å, respectively. Since the water bulk is placed between two brucite slabs, the regions greater than 10 Å and less than 10 Å are mirror-symmetrical, and both exhibit one high peak and two small peaks

**Figure 10:**
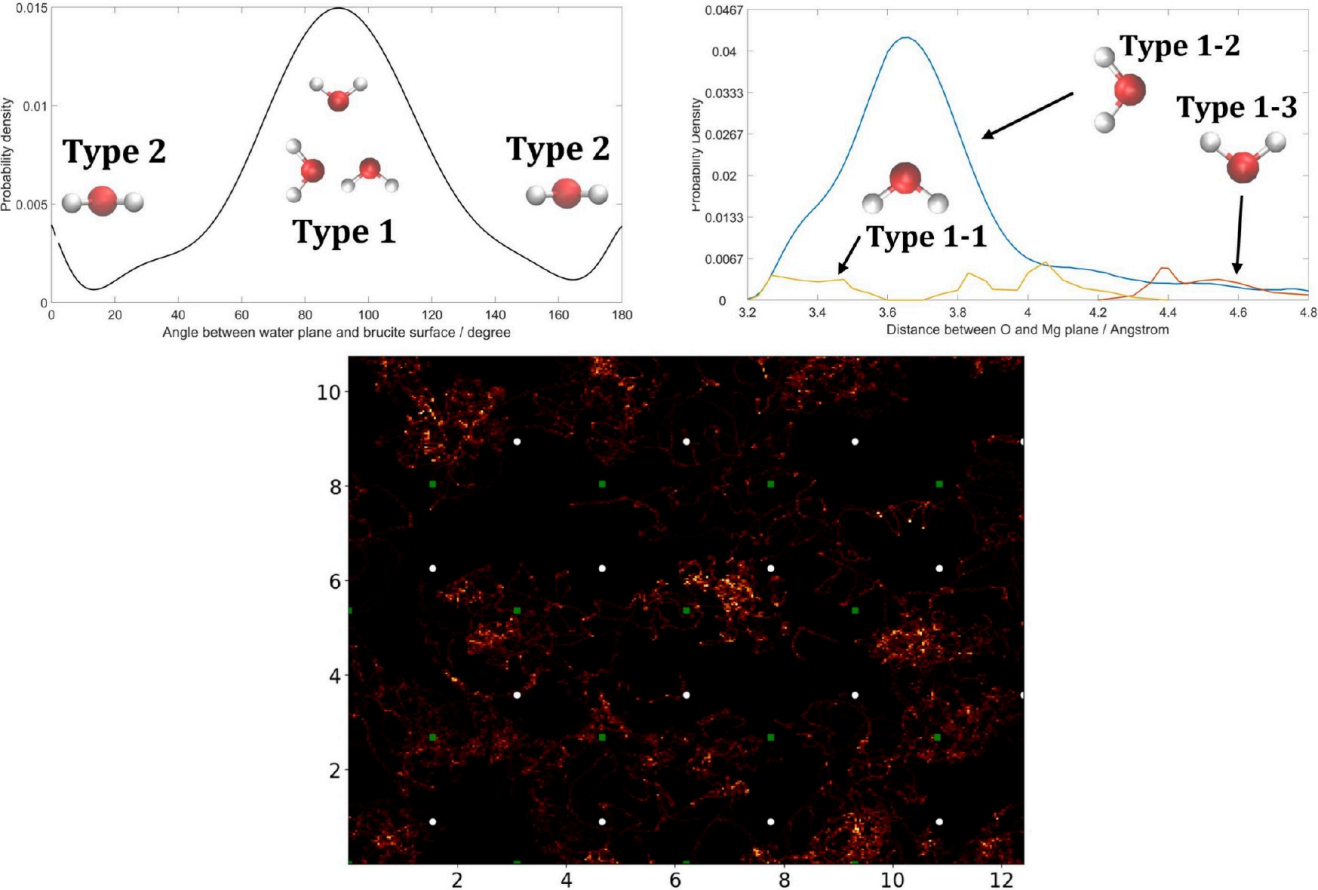
Bulk water on a brucite surface. (a) The density probability of water molecules with different geometries to the (0001) surface of brucite. Only water molecules with a distance between the O and the Mg plane less than 4.8 Å are considered. The curve is fitted and smoothed. (b) The density probability of water molecules with different angle between the water molecule plane and brucite(0001) surface. Only water molecules with the angle between water molecules and the brucite surface in the range of 75°–105° and the water molecules with a distance between the O and the magnesium surface less than 4.8 Å are considered. The curve for type 1-2 is fitted and smoothed, while the other two are not due to the small magnitude. (c) The probability map of H atom on the brucite(0001) surface. The figure is produced as an average over all the configurations following the energy becoming stable. High brightness represents a high probability of finding an H atom in a certain position, and low brightness represents a low probability. The white point is an OH group, and the green square is a Mg atom. Only water molecules whose distance between the O of the water molecule and the Mg atom is less than 4.8 Å are considered.

It is interesting to study the geometry of the water corresponding to the first peak as it may play an important role in hydration, dissolution, and the carbonation process. A distance of 4.8 Å is set as the cutoff, and the water molecules with a distance between O and Mg of less than 4.8 Å were studied. The water dipole moment direction is an important parameter that helps us to understand the geometry of water relative to the brucite surface in this system. Figure 10a shows the probability that a water molecule has a particular angle between its molecular plane and the (0001) surface. There are two obvious peaks, one at 90° and the other at 0/180°: the geometry of the water molecules is labeled on the curve. That is, the most favorable geometry for water molecules close to a brucite(0001) surface is with the dipole directly parallel and then perpendicular to the surface. In addition, there are more water molecules perpendicular to the (0001) surface than parallel to it. Figure 10b shows the probability density of water molecules dipole directions when dipole moments are perpendicular to the (0001) surface. We see that most of the water molecules have their dipoles parallel to the (0001) surface. The hydrogen bond between O in a water molecule and H in brucite may not be so strong because the number of molecules with their dipole direction along the normal of the (0001) surface is only  of the number of water molecules with their dipoles perpendicular to the surface’s normal. Water molecules with their dipoles directed toward the brucite(0001) surface are less likely to appear near the surface and only appear after 4.3 Å, while the water molecules with H atoms facing the surface vanish at ∼4.3 Å as well. Figure 10c shows the probability map of H atoms in water on the brucite(0001) surface. The H atoms are less likely to appear on the top of brucite OH groups and prefer to appear between two OH groups.

**Figure 11:**
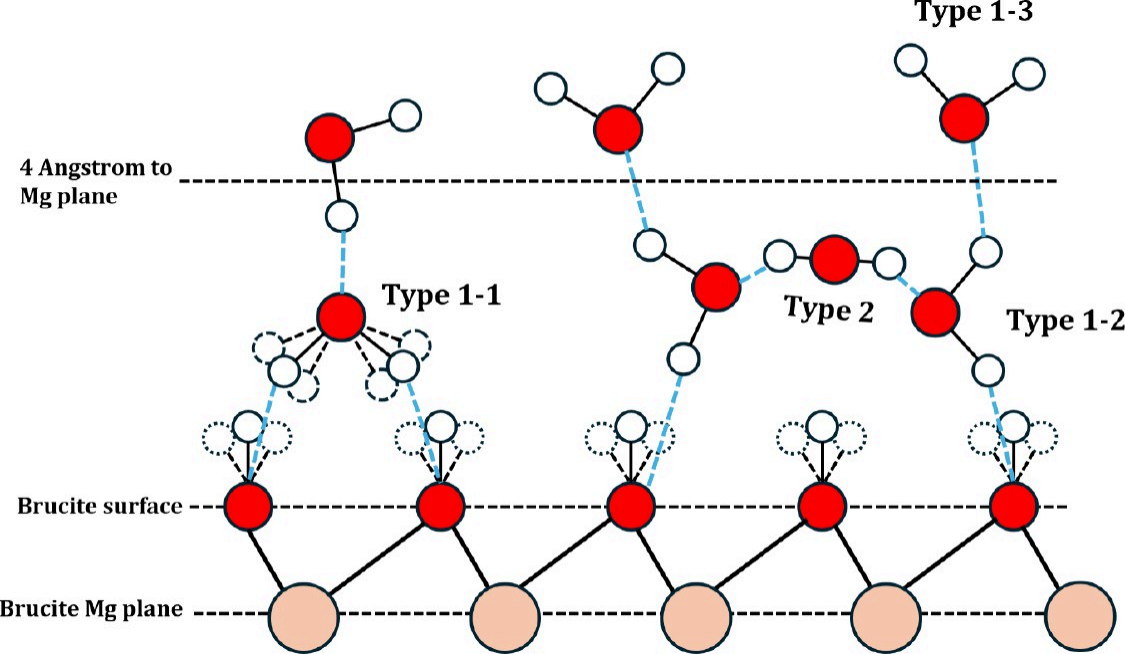
Schematic diagram for the near-surface water layer. The coral spheres are the magnesium atoms, the red spheres are the O atoms while the white spheres are the H atoms. The black solid and dash lines are the chemical bonds, while the blue dash lines are the hydrogen bonds.

From the information above, we can build a schematic diagram for the near-surface water layer, which is shown in Figure 11. First of all, there are two types of water molecules, which are those with their molecular plane being perpendicular to the surface plane (type 1) and those with their molecular plane being parallel to the surface plane (type 2). The number of type 1 molecules is greater than the number of type 2 ones. Type 2 molecules may play a role as a bridge to connect type 1 water molecules to other type 1 water molecules. In type 1 water molecules, the water molecules are further subdivided into three types, according to the dipole direction. They are the water molecule with dipole direction facing away from the brucite surface (type 1-1), with dipole direction parallel to the brucite surface (type 1-2), and with dipole direction toward the brucite surface (type 1-3). The number of type 1-2 water molecules is ∼5 times more than the other two types of water molecules. As a comparison, Wang et al.^65^ gave a similar brief near surface water structure of type 1–2 water molecules by classical force-field molecular dynamics. In addition, Ou et al.^66^ used CLAYFF force field for the same system and provided a similar first layer structure. However, their first layer does not contain type 1–1 water molecules, which is the difference between DFTB-MD and force field MD. Considering the low amount of type 1-1 water, it is possible that the structure emerged briefly after crossing the energy barrier.

## Conclusions

DFTB parameters for magnesium-related compounds were generated and applied in both static and DFTB-MD calculation. Our parameter set provided reliable geometry and electronic structures. Introducing dipole and quadrupole terms into *E*^(2)^ changes the electronic structure and partially alleviates the gap overestimation issues for the polarized system, but the absence of three-body and crystal field terms are still more important factors influencing the electronic structure of DFTB. Considering the calculation cost and convergence problems, only monopole and dipole terms are suitable to use. The incorporation of ELF and CDD into DFTB demonstrates their effectiveness as tools for electronic structure analysis, particularly in studying covalent bond formation. We note that calculations we performed with the 3ob parameter set show it can provide a description of adsorption with similar accuracy, even though it is designed for bio and organic system. However, the tools for electronic structure analysis including ELF and CDD using the 3ob parameter set remain to be developed.

When CO_2_ is adsorbed on the MgO(001) surface, Mg(OH)_2_(101̅1) surface and defective Mg(OH)_2_(0001) surface, CO_3_ group are formed on MgO(001) surface and defective Mg(OH)_2_(0001) surface, while CO_2_ remains physically adsorbed on the Mg(OH)_2_(101̅1) surface. When the CO_2_ is forced around a OH group on the Mg(OH)_2_(101̅1) surface with one of our earlier parameter sets, there is still no obvious covalent bond formed according to the ELF isosurface. This suggests that not only can DFTB describe the geometry but can also provide a reasonable description of the electronic structure. Moreover, the hydration process of MgO is weaker than its carbonation and even weaker for water molecules adsorbed on the Mg(OH)_2_(0001) surface.

For larger systems, the most favorable structures of bulk water on the brucite(0001) surface were also found. The hydrogen bond between an H atom in water molecules and an O atom in brucite plays a key role in the geometry around the brucite surface, which can be found in some classical MD simulations as well.^65,66^ However, water molecules with two hydrogen atoms pointing toward the brucite surface (type 1-1 H_2_O) are not found. This marks a clear difference between classical MD and DFTB-MD, but the proportion of this type of water molecule is far less than that of type 1-2 H_2_O. This should be further verified by ab initio MD. This is important, because the reconstruction of water near the surface may influence the dissolution and carbonation process. Further parametrization is needed to simulate carbon dioxide/carbonic acid solution and its reaction with brucite, and the physical adsorption problems on the Mg(OH)_2_(101̅1) surface.

One problem with our model is the energy of a system, especially for crystals: the energy changes during a reaction are overestimated. This may be solved by introducing a three-center term or a many-body potential into *E*^0^ in the future. But DFTB still remains a good choice for rapid selection of suitable structures providing a valuable starting point for further calculation in DFT, or an alternative solution when computational resources are limited.

## Data Availability

The DFTB parameters and the code to calculate the “*.cube” files for molecular orbitals (including HOMO and LUMO), ELF and CDD can be accessed via https://github.com/d3iven005/PLATO-tools. The “*.cube” file generated can be visualized by standard visualization software. Other data can be made available on request to the authors.

## Supporting Information Available

Details regarding DFTB parameterization; images depicting various structures for the Electron Localization Function; a comparison of different methods; MgO–CO_2_ and MgO–H_2_O adsorption calculations; Mg(OH)_2_–CO_2_ and Mg(OH)_2_–H_2_O adsorption calculations; the influence of introducing dipole and quadrupole terms; the energy of MgO–CO_2_ adsorption; MgO–H_2_O adsorption; Mg(OH)_2_–CO_2_ adsorption; and Mg(OH)_2_–H_2_O adsorption (PDF)
Three-dimensional image showing the atomic motion of CO_2_ molecule adsorption on a defective Mg(OH)_2_ (0001) surface (MP4)
