# Supplementary material for: Tight Binding Simulation of the MgO and Mg(OH)2 Hydration and Carbonation Processes
Source: J Chem Theory Comput. 2025 Feb 4;21(4):1961–77. doi: 10.1021/acs.jctc.4c01531 (PMC11875438; doi:10.1021/acs.jctc.4c01531)
Supplement: Supplementary file 1 — ct4c01531_si_001.pdf [file ct4c01531_si_001.pdf]

Supporting Information of Tight binding simulation of the MgO and $Mg(OH)_2$ hydration and carbonation processes

Jiwen Yu and Andrew Horsfield*

Department of Materials and Thomas Young Centre, Imperial College London, South Kensington Campus, London SW7 2AZ, United Kingdom

E-mail: a.horsfield@imperial.ac.uk

DFTB parameterisation

Table S1: The onsite energies and Hubbard parameters for H, C, O and Mg

Element	Orbitals	ϵ_s/Ry	ϵ_p/Ry	U/Ry
H	$1s^1$	-0.4381		0.8990
C	$2s^22p^2$	-1.0109	-0.3880	0.7625
O	$2s^22p^4$	-1.7349	-0.6355	1.0437
Mg	$3s^22p^0$	-0.3452	-0.0978	0.5645

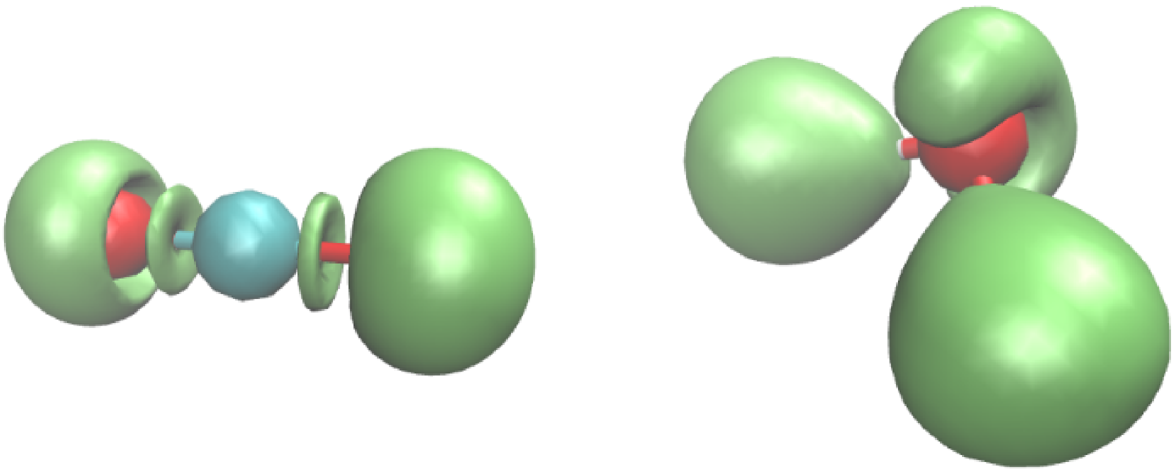

Figure S1: the ELF isosurface with a value of 0.8 of CO_2 (left) and H_2O (right) calculated by PLATO.

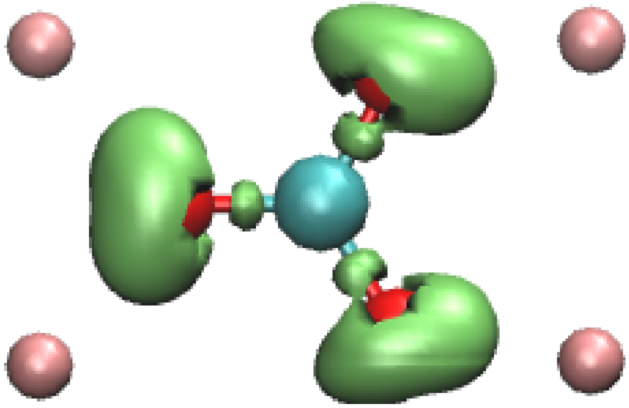

Figure S2: the ELF isosurface with a value of 0.8 of CO_3 group in $MgCO_3$.

Comparison of different methods

Lattice constant

Table S2: The optimised lattice constant and band gap of MgO and $Mg(OH)_2$ using different methods in angstrom

	MgO		$Mg(OH)_2$	
	Lattice constant	Band gap	Lattice constant	Band gap
PBE	4.25	4.64	a=3.18 c=4.59	4.46
PBEsol	4.21	4.99	a=3.14 c=4.54	4.68
HSE06	4.21	6.95	a=3.24 c=4.67	7.18
3ob	4.31	7.55	a=3.17 c=4.36	10.70
PTBP	4.06	5.34	a=2.96 c=4.05	8.12
MATSCI	4.23	10.94	a=3.21 c=4.18	11.54
PLATO	4.25	8.53	a=3.10 c=4.58	9.95
experiment[1,2]	4.21	7.80	a=3.15 c=4.77	5.70

Surface

Table S3: The optimised surface structure of MgO and $Mg(OH)_2$. Δ is the difference of lattice constant (based on Mg) between the (first layer - second layer) and (second layer - third layer), or the vertical distance between Mg and O in MgO. - represents the distance is smaller than that of bulk, and + represents larger.

	Δ(MgO)	Δ(Mg-O (MgO))	Δ($Mg(OH)_2$ (0001))
PBE	0.017	0.05	0.015
PBEsol	0.019	0.05	0.002
3ob	0.066	0.10	0.000
PTBP	0.064	0.09	0.008
MATSCI	0.008	0.13	0.004
PLATO	0.002	0.02	0.002

$MgO-CO_2$ and $MgO-H_2O$ adsorption calculations

Table S4: The optimised C-O bond length and O-C-O bond angle of CO_2 adsorbed on MgO (001) surface.

	$C(CO_2)$- $O(CO_2)$	$C(CO_2)$- O(MgO)	$\angle O(CO_2)-O(CO_2)-C(CO_2)$
PBE	1.25	1.47	133.7
PBEsol	1.25	1.44	133.0
HSE06	1.24	1.44	133.4
3ob	1.27	1.39	128.6
PTBP	1.29	1.38 (CO_3 tilted)	131.4
PLATO	1.25	1.43	135.8

For the H_2O adsorbed on the MgO surface, because the configuration difference is large, the comparison is shown in figure S4.

$Mg(OH)_2-CO_2$ and $Mg(OH)_2-H_2O$ adsorption calculations

Table S5: The data calculated from the optimised CO_2 and H_2O adsorbed on $Mg(OH)_2$ $(10\bar{1}1)$ surface by different methods.

	d(C-surface)	$d(H(H_2O)$ - $O(Mg(OH)_2))$
PBE	3.13	1.62
PBEsol	2.71	1.68
HSE06	2.82	1.86
3ob	2.26	1.76
PTBP	0.79	1.68
PLATO	2.18	1.82

Table S6: The data of optimized CO_2 adsorbed on defective $Mg(OH)_2$ (0001) surface.

	$\angle CO_3$ - (0001) surface	C - O (bottom)	C - O (top)	$\angle O(CO_2)-C(CO_2)-O(CO_2)$
3ob	71.6	1.35	1.26	122.1
PTBP	59.9	1.36	1.28	123.4
PLATO	68.8	1.37	1.24	122.3

The structure of the data in the table above.

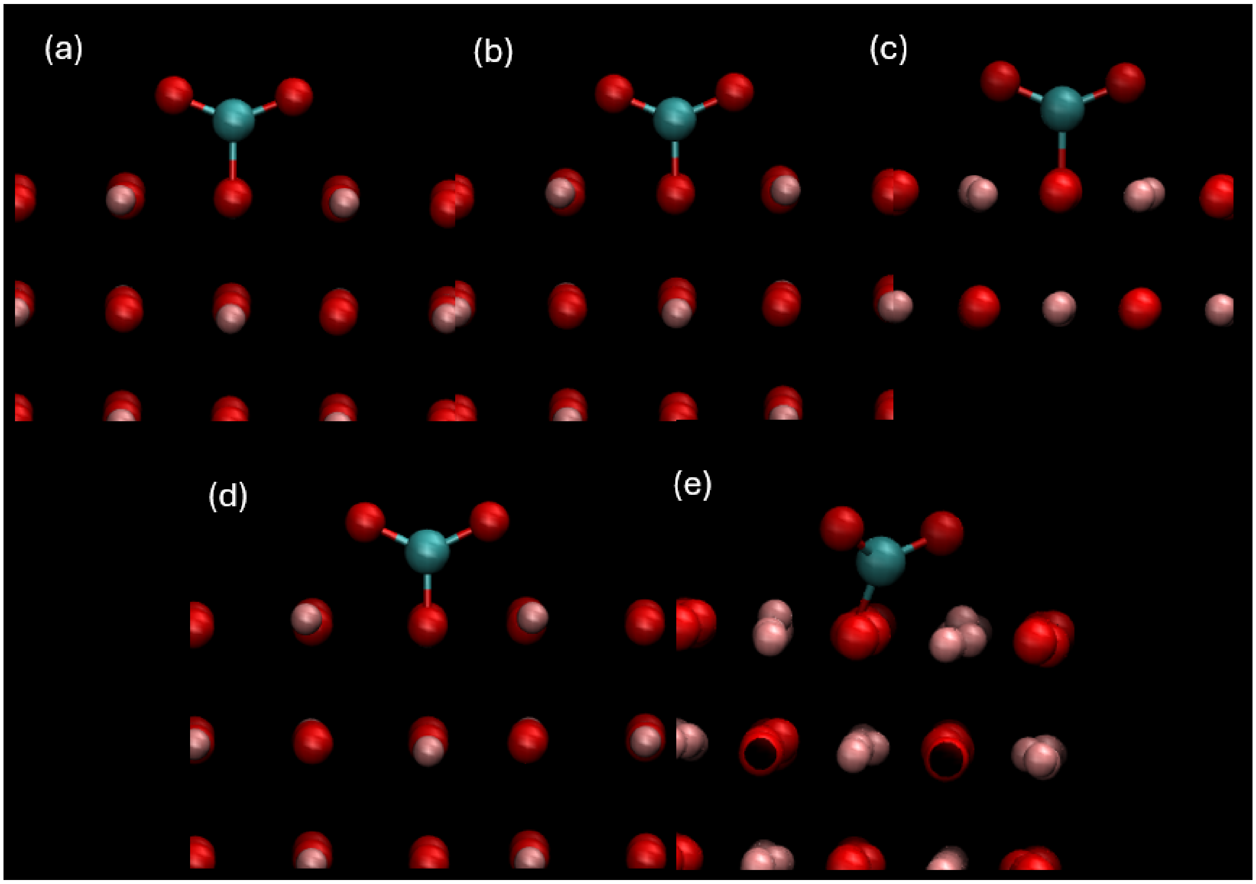

Figure S3: The optimised structure of CO_2 adsorbed on the (001) surface of MgO calculated by (a) PBE-DFT, (b) PBEsol-DFT, (c) HSE06-DFT, (d) 3ob-DFTB and (e)PTBP-DFTB.

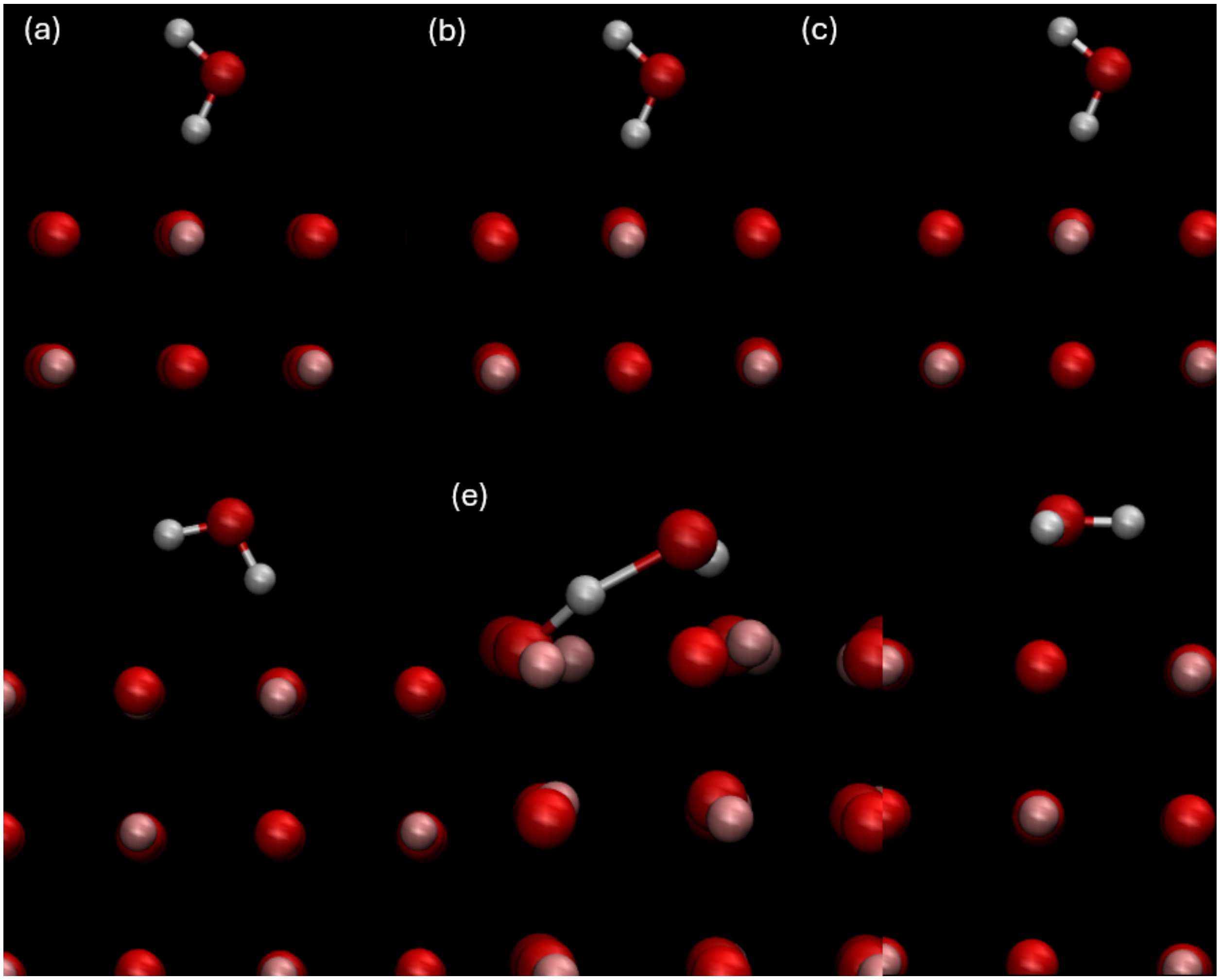

Figure S4: The optimised structure of H_2O adsorbed on the (001) surface of MgO calculated by (a) PBE-DFT, (b) PBEsol-DFT, (c) HSE06-DFT, (d) 3ob-DFTB , (e)PTBP-DFTB and (f) matsci.

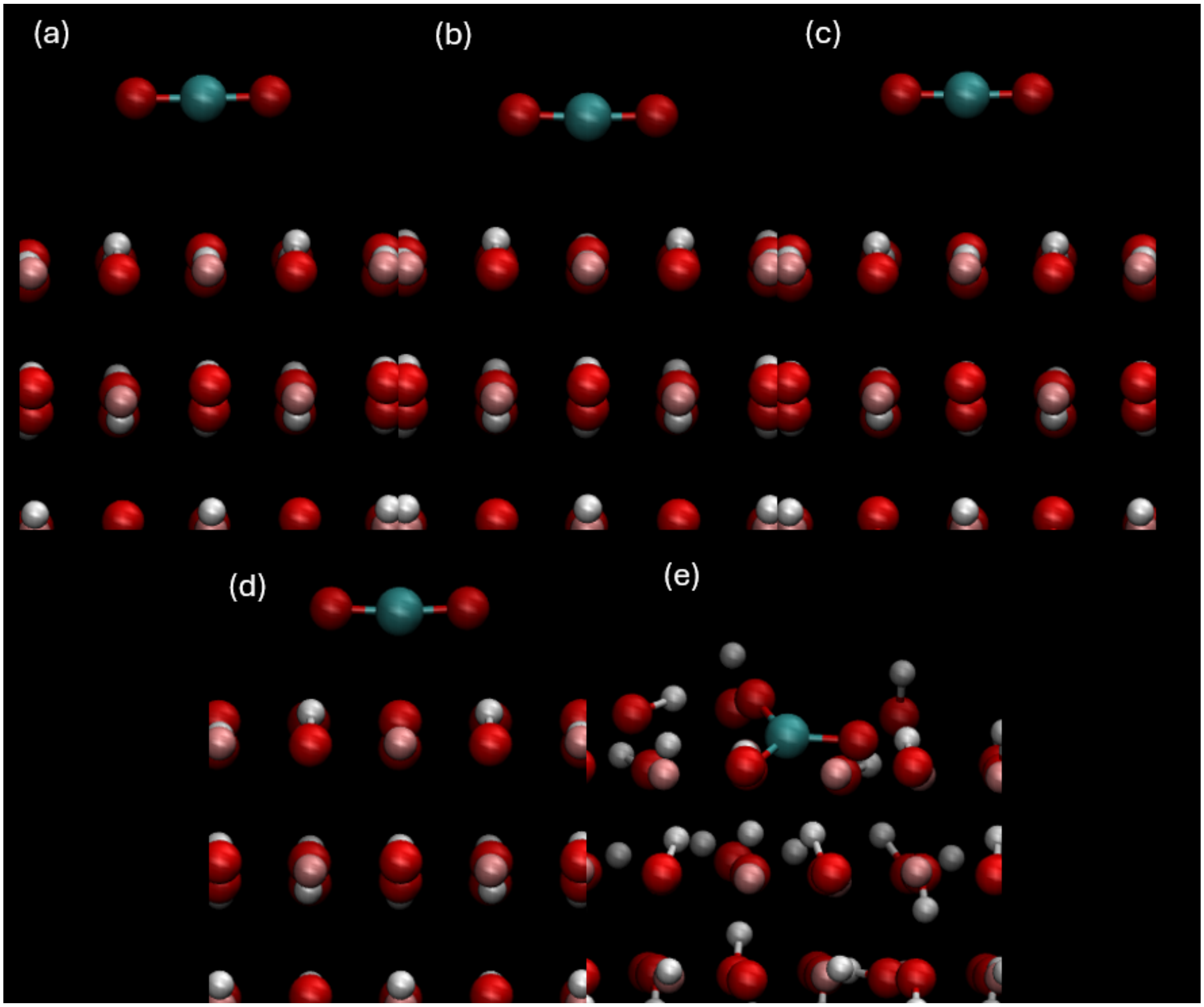

Figure S5: The optimised structure of CO_2 adsorbed on the $(10\bar{1}1)$ surface of $Mg(OH)_2$ calculated by (a) PBE-DFT, (b) PBEsol-DFT, (c) HSE06-DFT, (d) 3ob-DFTB and (e)PTBP-DFTB.

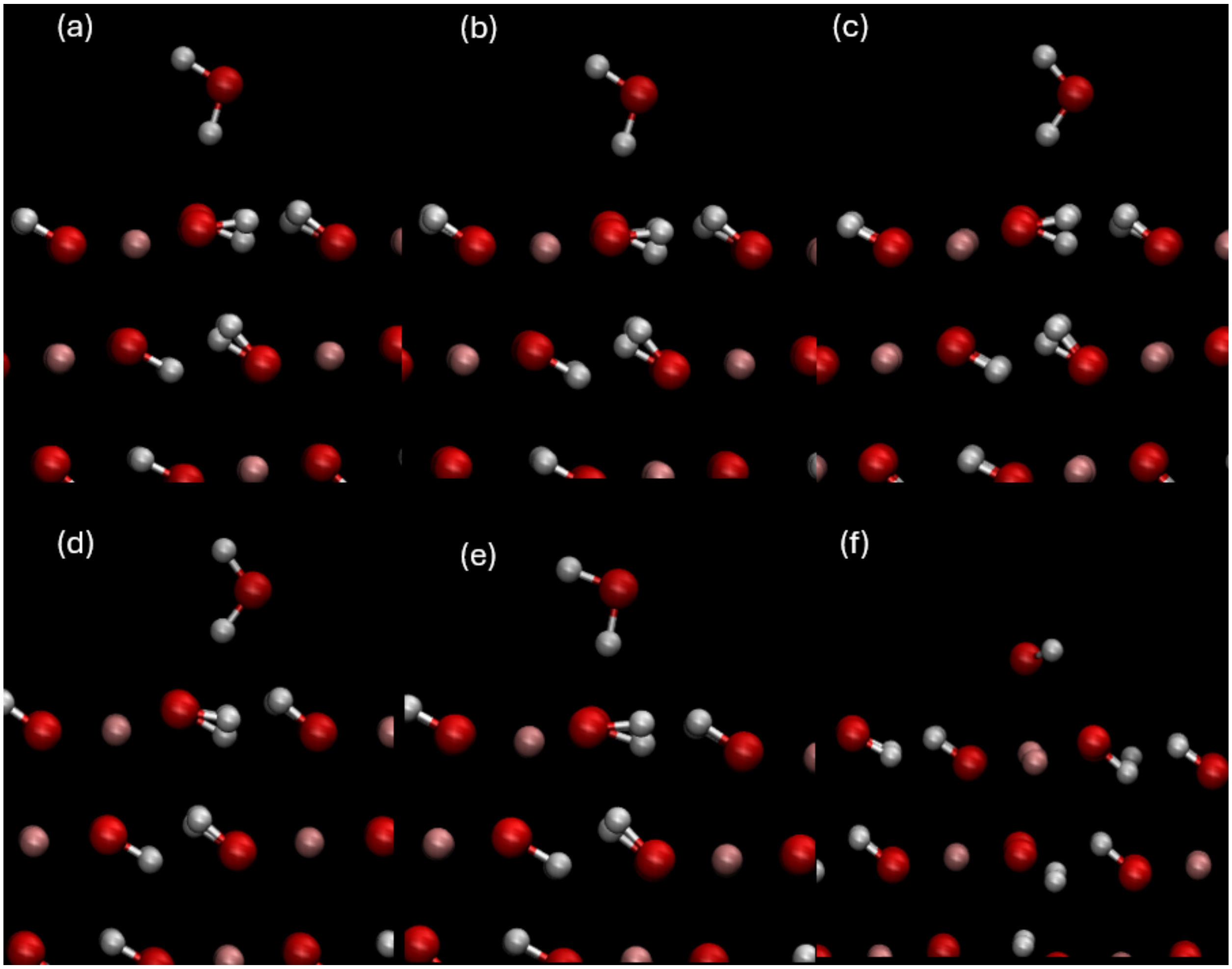

Figure S6: The optimised structure of H_2O adsorbed on the (10$\bar{1}$1) surface of $Mg(OH)_2$ calculated by (a) PBE-DFT, (b) PBEsol-DFT, (c) HSE06-DFT, (d) 3ob-DFTB , (e)PTBP-DFTB and (f) matsci.

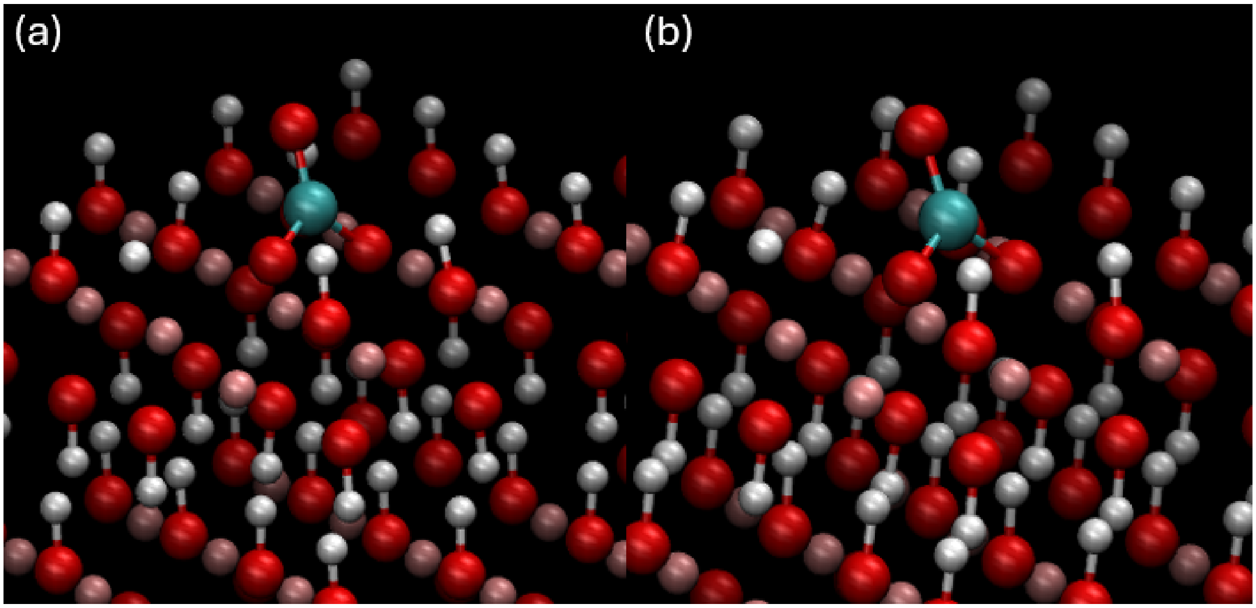

Figure S7: The optimised structure of CO_2 adsorbed on the defective (0001) surface of $Mg(OH)_2$ calculated by (a) 3ob-DFTB and (e)PTBP-DFTB

The influence of introducing dipole and quadrupole terms

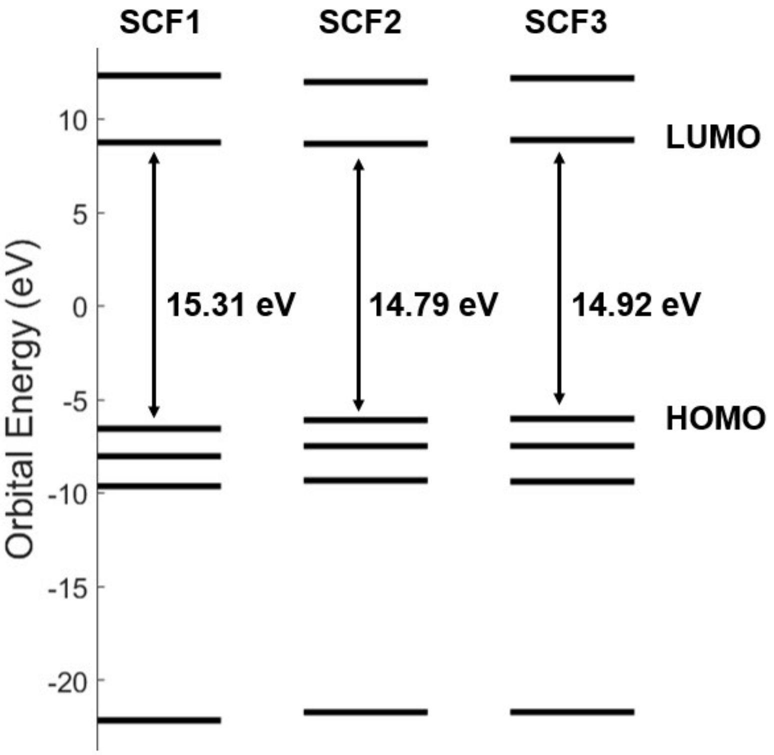

Figure S8: The orbital energy levels of a water molecule by DFTB with three self-consistent schemes: monopole only (SCF1), monopole and dipole (SCF2), and monopole, dipole and quadrupole (SCF3).

Figure S8 shows the energy level for water molecule and table S7 shows the detailed contribution of each atomic orbital calculated by SCF1, SCF2 and SCF3.

Table S7: The energy levels and the coefficients for each orbitals of H_2O calculated by SCF1, SCF2 and SCF3, the dipole direction is toward x-axis.

	SCF1	SCF2	SCF3
**Energy Level**	**-22.1668**	**-21.7288**	**-21.723**
O_s	0.8916542154	0.8892659439	0.888434874
O_{px}	0	0	0
O_{py}	-0.01902384506	0.01945986033	0.02031192299
O_{pz}	0	0	0
H_s1	0.1266120426	0.1268085881	0.1270843188
H_s2	0.1266120426	0.1268085881	0.1270843188
**Energy Level**	**-9.63742**	**-9.33062**	**-9.38941**
O_s	0	0	0
O_{px}	-0.6638081476	0.6511311093	-0.6514049592
O_{py}	0	0	0
O_{pz}	0	0	0
H_s1	-0.3530790973	0.3644586977	-0.3631441435
H_s2	0.3530790973	-0.3644586977	0.3631441435
**Energy Level**	**-8.03413**	**-7.49255**	**-7.48544**
O_s	0.2631575277	0.3000739185	0.301192854
O_{px}	0	0	0
O_{py}	-0.8357565803	-0.8310556205	-0.8320664486
O_{pz}	0	0	0
H_s1	-0.2314854165	-0.2314886302	-0.2308395258
H_s2	-0.2314854165	-0.2314886302	-0.2308395258
**Energy Level**	**-6.56439**	**-6.10789**	**-6.03344**
O_s	0	0	0
O_{px}	0	0	0
O_{py}	0	0	0
O_{pz}	-1	-1	1
H_s1	0	0	0
H_s2	0	0	0
**Energy Level**	**8.74857**	**8.67773**	**8.88351**
O_s	0	0	0
O_{px}	-0.9642234911	-0.9691918521	0.9726457611
O_{py}	0	0	0
O_{pz}	0	0	0
H_s1	0.7881669311	0.7806489608	-0.7835804306
H_s2	-0.7881669311	-0.7806489608	0.7835804306
**Energy Level**	**12.3253**	**11.9811**	**12.181**
O_s	-0.7679821771	0.7525362274	-0.7576688511
O_{px}	0	0	0
O_{py}	-0.7273697777	0.7330551788	-0.731553561
O_{pz}	0	0	0
H_s1	0.8414932566	-0.8395715407	0.8415994876
H_s2	0.8414932566	-0.8395715407	0.8415994876

MgO-CO_2 Adsorption

Figure S9 shows the starting points for CO_2 to adsorb on, and table S8 shows the final adsorption energy after optimisation at different starting point. The name for the starting geometry is "<molecule in CO_2>_on_<the position on MgO surface>_direction index". The CO_2 has two starting geometries, which are parallel to the surface plane (index starting with 0 in table S8) and perpendicular to the surface plane (index starting with 1 in table S8). The parallel in the table means that the CO_2 molecule direction is along Mg-O bond, while 45 represents that the angle between CO_2 molecule direction and Mg-O bond is 45°.

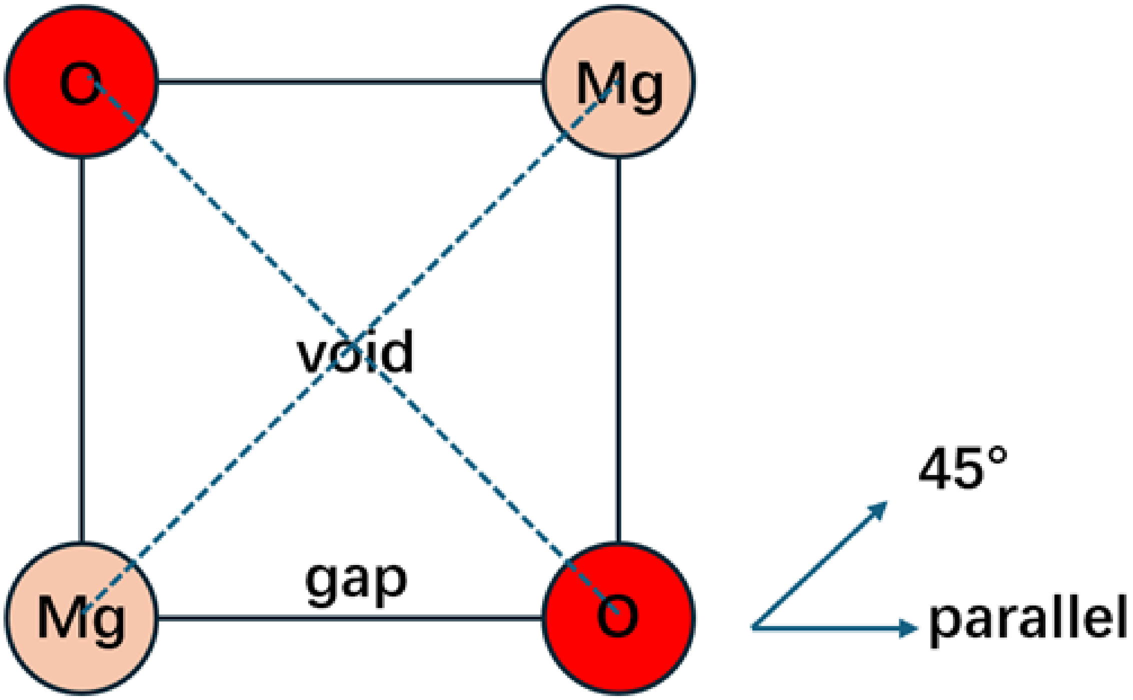

Figure S9: The simplified structure of MgO (001) surface, the high symmetric starting points are Mg atom, O atom, the midpoint of Mg-O bond(gap), and the void. The axis shows the direction of molecules.

Table S8: The adsorption energy for CO_2 at different starting points.

Index	Starting geometry	Adsorption energy /Ry
01	C_on_Mg_parallel	-0.2809
02	C_on_Mg_45	-0.3319
03	C_on_O_parallel	-0.6329
04	C_on_O_45	-0.6329
05	O_on_Mg_parallel	-0.5190
06	O_on_Mg_45	-0.3399
07	C_on_gap_parallel	-0.5890
08	C_on_void_parallel	-0.3340
09	C_on_void_45	-0.2050
11	O_on_Mg	-0.4175
12	O_on_O	-0.1960
13	O_on_gap	-0.1834
14	O_on_void	-0.3206

MgO-H_2O Adsorption

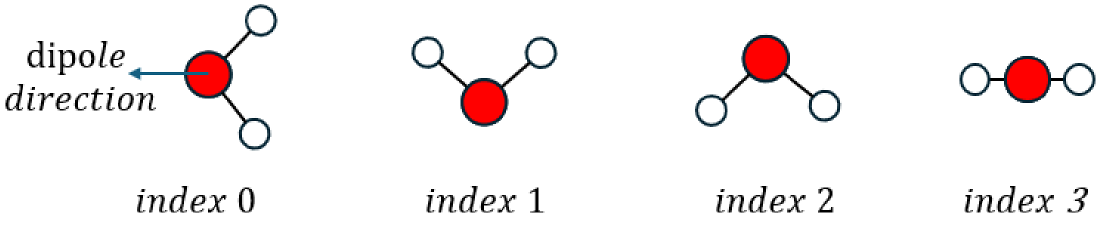

Figure S10: The water molecule geometry for different starting point.

Figure S10 shows the different starting geometries of water molecules, and the first number in table S9 represents the index number showing in the figure. The name for the starting geometry is "<molecule in H_2O>_on_<the position on MgO surface>_direction index". The direction-index indicates the direction in which the dipole moment of the water molecules is oriented. The 'parallel' in the table means that the dipole direction of H_2O molecule direction is along Mg-O bond, while '45' represents that the angle between H_2O dipole direction and Mg-O bond is 45°. 90 only appears when the molecule is placed on the gap, and it means the dipole direction is toward the intersection of two blue dash lines.

Table S9: The adsorption energy for H_2O at different starting points.

Index	Starting geometry	Adsorption energy /Ry
01	H_on_O_parallel	-0.3151
02	H_on_O_45	-0.4005
03	H_on_Mg_parallel	-0.2064
04	H_on_Mg_45	-0.2385
05	H_on_gap_parallel	-0.3939
06	H_on_gap_90	-0.2345
07	H_on_void_parallel	-0.1830
08	H_on_void_45towardsMg	-0.2152
11	O_on_O_parallel	-0.1428
12	O_on_O_45	-0.0267
13	O_on_Mg_parallel	-0.1903
14	O_on_Mg_45	-0.3779
15	O_on_gap_parallel	-0.2137
16	O_on_gap_90	-0.2006
17	O_on_void_parallel	-0.2512
18	O_on_void_45twdO	-0.0454
19	O_on_void_45twdMg	-0.1187
21	O_on_O_parallel	-0.3059
22	O_on_O_45	-0.3295
23	O_on_Mg_parallel	-0.2044
24	O_on_Mg_45	-0.2715
25	O_on_gap_parallel	-0.3406
26	O_on_gap_90	-0.1906
27	O_on_void_parallel	-0.3758
28	O_on_void_45twdO	-0.2283
29	O_on_void_45twdMg	-0.2123

$Mg(OH)_2$-CO_2 Adsorption

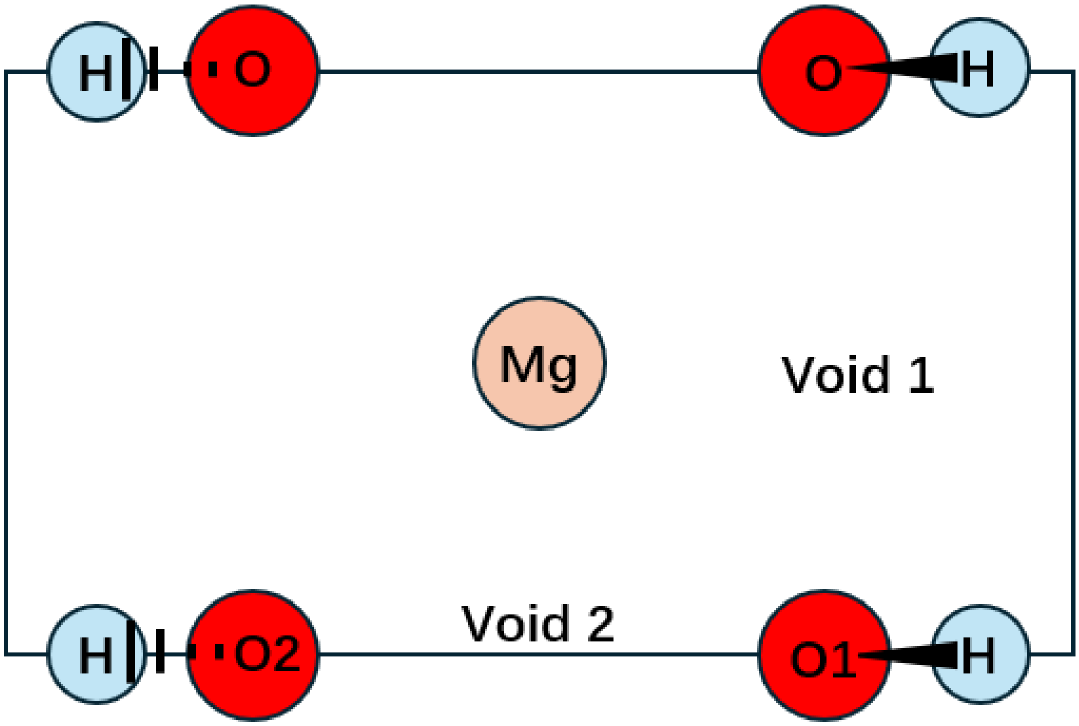

Figure S11: The simplified structure of $Mg(OH)_2$ $(10\bar{1}1)$ surface, the starting points are Mg atom, O1 and O_2 atom, and the two voids. The illustration is not drawn to scale.

Figure S11 shows the simplified structure of $Mg(OH)_2$ surface and the starting points are Mg, O and two voids. The solid triangle lines for OH bonds refers to the hydrogen atoms being above the paper plane, while the dash triangle lines refers to the hydrogen atoms being below the paper plane. The name for the starting geometry is "<molecule in CO_2>_on_<the position on $Mg(OH)_2$ surface>_direction index". Table S10 shows the final adsorption energy for each starting point. The index starting with 0 indicates that the CO_2 molecule is parallel to the surface plane, while the index starting with 1 indicates that the molecule is perpendicular to the surface plane. 'parallel' refers to the molecule being aligned along the direction of the line connecting O1 and O2, while '90' represents the molecule is perpendicular

to the line connecting O1 and O2.

Table S10: The adsorption energy for CO_2 at different starting points.

Index	Starting geometry	Adsorption energy /Ry
01	C_on_O1_paralle	-0.2577
02	C_on_O1_90	-0.2849
03	C_on_O2_parallel	-0.1698
04	C_on_O2_90	-0.2426
05	C_on_Mg_parallel	-0.1408
06	C_on_Mg_90	-0.2065
07	C_on_V1_parallel	-0.1420
08	C_on_V1_90	-0.1164
09	C_on_V2_parallel	-0.2808
010	C_on_V2_90	-0.2924
11	O_on_O1	-0.1763
12	O_on_O2	-0.0961
13	O_on_Mg	-0.1232
14	O_on_V1	-0.1291
15	O_on_V2	-0.0907

$Mg(OH)_2$-H_2O Adsorption

Table S11 shows the final adsorption energy for each starting point. The name for the starting geometry is "<molecule in H_2O>_on_<the position on $Mg(OH)_2$ surface>_direction index". The first index represents the situations in figure S10. The direction-index indicates the dipole direction of H_2O. In addition, '+' represents that the dipole direction is toward the O1, while '-' represents that the dipole direction is toward the O2.

Table S11: The adsorption energy for H_2O at different starting points.

Index	Starting geometry	Adsorption energy /Ry
01	H_on_O1_parallel_+	-0.1428
02	H_on_O1_parallel_-	-0.0676
03	H_on_O1_90	-0.1511
04	H_on_O2_parallel_+	-0.0167
05	H_on_O2_parallel_-	-0.1247
06	H_on_O2_90	-0.1389
07	H_on_Mg_parallel	-0.1128
08	H_on_Mg_90	-0.1189
09	H_on_V1_parallel	-0.1245
010	H_on_V1_90	-0.0987
011	H_on_V2_parallel	-0.1156
012	H_on_V2_90	-0.1636
11	O_on_Mg_parallel	-0.1172
12	O_on_Mg_90	-0.1053
13	O_on_O1_parallel	-0.1491
14	O_on_O1_90	0.2140
15	O_on_V1_parallel	-0.1031
16	O_on_V1_90	-0.1042
17	O_on_V2_parallel	-0.1038
18	O_on_V2_90	-0.1169
21	O_on_Mg_parallel	-0.0563
22	O_on_Mg_90	-0.1039
23	O_on_O1_parallel	-0.1279
24	O_on_O1_90	0.2140
25	O_on_V1_parallel	-0.0920
26	O_on_V1_90	-0.0435
27	O_on_V2_parallel	-0.1019
28	O_on_V2_90	-0.1075

References

(1) Heo, S.; Cho, E.; Lee, H.-I.; Park, G. S.; Kang, H. J.; Nagatomi, T.; Choi, P.; Choi, B.-D. Band gap and defect states of MgO thin films investigated using reflection electron energy loss spectroscopy. *AIP Advances* **2015**, *5*.

(2) Kumari, L.; Li, W.; Vannoy, C. H.; Leblanc, R. M.; Wang, D. Synthesis, characterization and optical properties of Mg (OH) 2 micro-/nanostructure and its conversion to MgO. *Ceramics International* **2009**, *35*, 3355–3364.
